# Mannosylated brush copolymers based on poly(ethylene glycol) and poly(ε-caprolactone) as multivalent lectin-binding nanomaterials

**DOI:** 10.3762/bjnano.10.212

**Published:** 2019-11-07

**Authors:** Stefania Ordanini, Wanda Celentano, Anna Bernardi, Francesco Cellesi

**Affiliations:** 1 Department of Chemistry, Materials and Chemical Engineering “G. Natta”, Politecnico di Milano, via Mancinelli 7, Milano 20131, Italy; 2 Humanitas Research Hospital, Via Manzoni 56, Rozzano, Milano 20089, Italy; 3 Department of Chemistry, Università degli Studi di Milano, via Golgi 19, Milano 20133, Italy

**Keywords:** atom transfer radical polymerization (ATRP), glycopolymer, lectin, poly(ethylene glycol), poly(ε-caprolactone), ring-opening polymerization (ROP)

## Abstract

A class of linear and four-arm mannosylated brush copolymers based on poly(ethylene glycol) and poly(ε-caprolactone) is presented here. The synthesis through ring-opening and atom transfer radical polymerizations provided high control over molecular weight and functionality. A post-polymerization azide–alkyne cycloaddition allowed for the formation of glycopolymers with different mannose valencies (1, 2, 4, and 8). In aqueous media, these macromolecules formed nanoparticles that were able to bind lectins, as investigated by concanavalin A binding assay. The results indicate that carbohydrate–lectin interactions can be tuned by the macromolecular architecture and functionality, hence the importance of these macromolecular properties in the design of targeted anti-pathogenic nanomaterials.

## Introduction

Carbohydrate–protein interactions are involved in many biological processes, including cell recognition and cell–cell adhesion. These interactions drive pathological events, such as cellular infections by viruses (e.g., HIV and Ebola [1–2]) and toxins (e.g., Shiga and Cholera toxins [3]). Carbohydrate–protein interactions in biological systems are mostly multivalent, which allows one to enhance their strength with respect to the weak single saccharide–protein connections. Carbohydrate-binding proteins are known as lectins. A way to interfere with pathological carbohydrate–protein interactions is the use of artificial ligands able to antagonize lectins, possibly with higher affinity than the natural ligands. Multivalent glycoconjugates have been recently synthesized with the aim of producing powerful anti-pathogenic agents [4]. The so called “cluster glycoside effect” is the enhanced activity of a multivalent glycoderivative with respect to the monovalent saccharide [5].

Glycopolymers, i.e., polymers presenting pendant saccharides, are an important and well-studied class of synthetic glycoclusters [6]. They have the advantage of being easy to synthesize and present larger valencies with respect to other multivalent compounds, thus exhibiting an amplified binding potency towards lectins. In some cases, glycopolymers have shown affinities for lectins that are comparable to those of antigens and antibodies [7]. Nevertheless, polymers often lack homogeneity; they can be highly polydisperse and poorly characterized, and this represents a clear limitation for therapeutic applications, since different molecular weight, architecture and functionality can influence the macromolecule bioactivity [8]. Controlled radical polymerization (CRP) techniques, such as atom transfer radical polymerization (ATRP), reversible addition fragmentation chain transfer (RAFT) polymerization, single-electron transfer living radical polymerization (SET-LRP) and nitroxide-mediated radical polymerization (NMP), are often used to produce well-defined glycopolymers with controlled molecular weight and narrow molecular weight distribution [9–10]. In particular, ATRP is a living radical polymerization catalyzed by a transition metal, normally copper or ruthenium, and initiated by halogenated compounds, typically alkyl halides. ATRP is tolerant of a large range of functional groups; the use of multifunctional initiators allows for the synthesis of materials with different topologies, e.g., comb-like and star polymers [11].

ATRP of an unprotected carbohydrate-functionalized monomer, starting from a four-arm initiator based on poly(ε-caprolactone), led to a library of glucose-functionalized aggregates able to bind the glucose- and mannose-binding lectin Concanavalin A (Con A) [12]. The combination of ATRP of protected alkyne monomers and a copper-catalyzed azide–alkyne click cycloaddition (CuAAC) with azide-bearing sugars was reported as an effective strategy to prepare multivalent mannose- and galactose-pendant polymers. Their interaction with Con A strictly depended on the mannose epitope density: fully mannosylated polymers were more active than the corresponding partially galactose-functionalized molecules [13]. Analogous copolymers were tested as ligands for DC-SIGN, a tetravalent lectin involved in the early stage of HIV infection [1]. Also in this case, molecules presenting the highest mannose density had the highest activity towards the lectin [14]. A class of five-arm and eight-arm glycopolymers bound to DC-SIGN with picomolar affinities, one order of magnitude more than the corresponding linear molecules. The authors speculated that this may occur because of the ability of star-shaped polymers to bind simultaneously the four lectin carbohydrate-recognition domains [15]. DC-SIGN was efficiently targeted also by mannose-decorated polymers synthesized through SET-LRP of glycomonomers [16]. When amphiphilic glycopolymers were produced, they formed nanoparticles in aqueous solution, and their binding activities were also affected by shape and size [17]. In addition, the high surface area of spherical self-assembled structures can confer glycopolymers a high affinity towards lectins [18].

Among different synthetic macromolecules used in biomedical applications, copolymers based on poly(ethylene glycol) (PEG) and poly(ε-caprolactone) (PCL) are found in many FDA-approved products [19]. PCL repeating units are constituted by five non-polar methylene groups and one labile ester group. It was reported that biodegradability and performance of PCL in cell culture studies are enhanced when it is modified with PEG [20]. PEG is a hydrophilic nontoxic polymer that does not promote immune responses. Pegylation reduces the nonspecific binding of nanoparticles to blood proteins and macrophages, making them non-immunogenic and non-antigenic [21].

PEG-PCL copolymers are amphihilic materials that can spontaneously assemble in aqueous media, forming colloidal aggregates (e.g., micelles and vesicles) above their critical aggregation concentration (CAC). The resulting self-assembled nanoparticles can act as drug carriers and delivery systems, being able to accommodate a hydrophobic drug within their hydrophobic core [22], or chemically bind bioactive agents [23–24].

**Figure 1:**
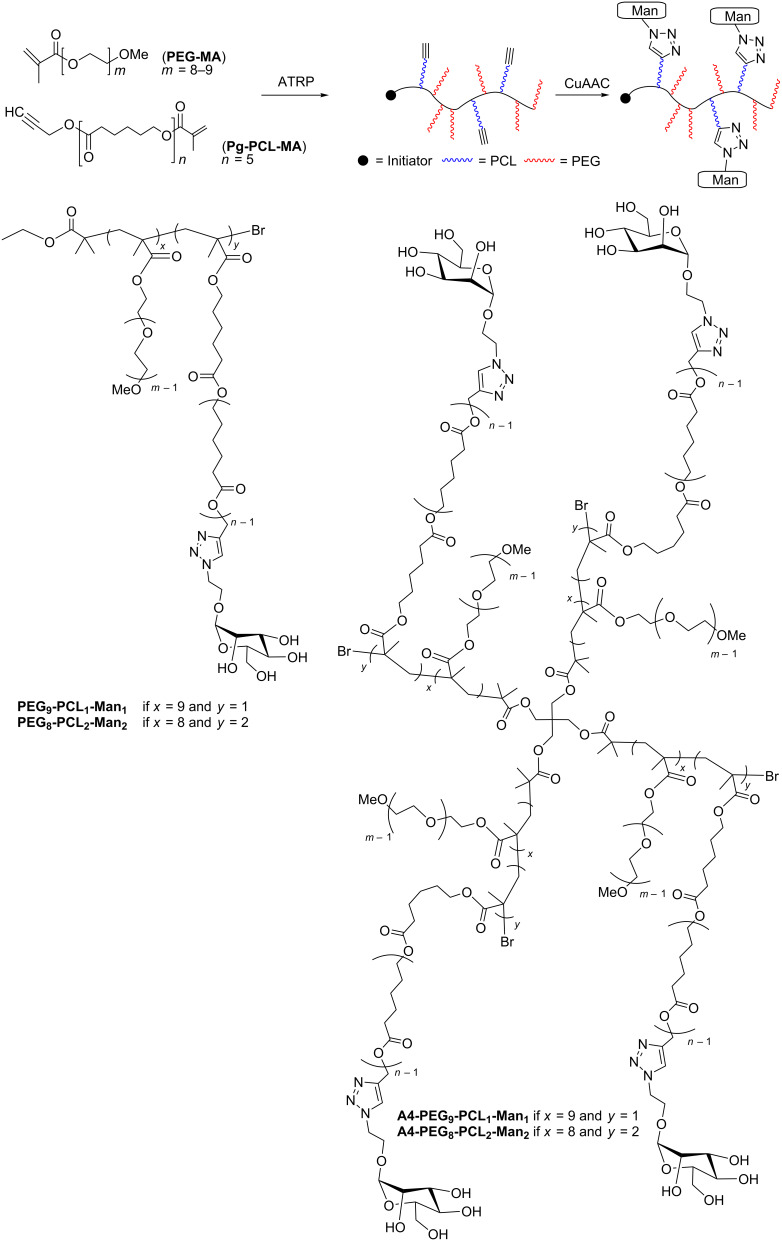
Synthetic approach (upper panel), final structure and nomenclature (lower panels) of the mannose-functionalized PEG-PCL copolymers.

In this work, we used ATRP to produce a library of brush copolymers, which were synthesized via random copolymerisation of PEG- and PCL-based macromonomers. Their polymeric backbones were then mannosylated through a post-polymerization CuAAC, in order to obtain potential lectin ligands. Copolymers of different shapes were synthesized, i.e., linear or four-arm, according to the initiator used for the ATRP. The percentage of PCL blocks bearing a triple bond with respect to the PEG blocks was varied. Different clickable polymeric backbones were produced and finally mannosylated, obtaining different overall mannose valencies (1, 2, 4, and 8). The structures, acronyms and valencies of the synthesized comb-like-glycopolymers are reported in Figure 1. The final glycopolymers have a flexible structure that should confer them the possibility to freely adjust the position of the mannose residues, matching the one of the lectin binding sites. Their capability to effectively expose mannose and behave as lectin ligands was investigated using Concanavalin A as a model lectin. Moreover, the glycopolymers are amphiphilic materials, and their ability to spontaneously self-assemble in nano-sized-aggregates was also examined.

## Results and Discussion

### Synthesis of the PCL-based macromonomer

**Scheme 1:**
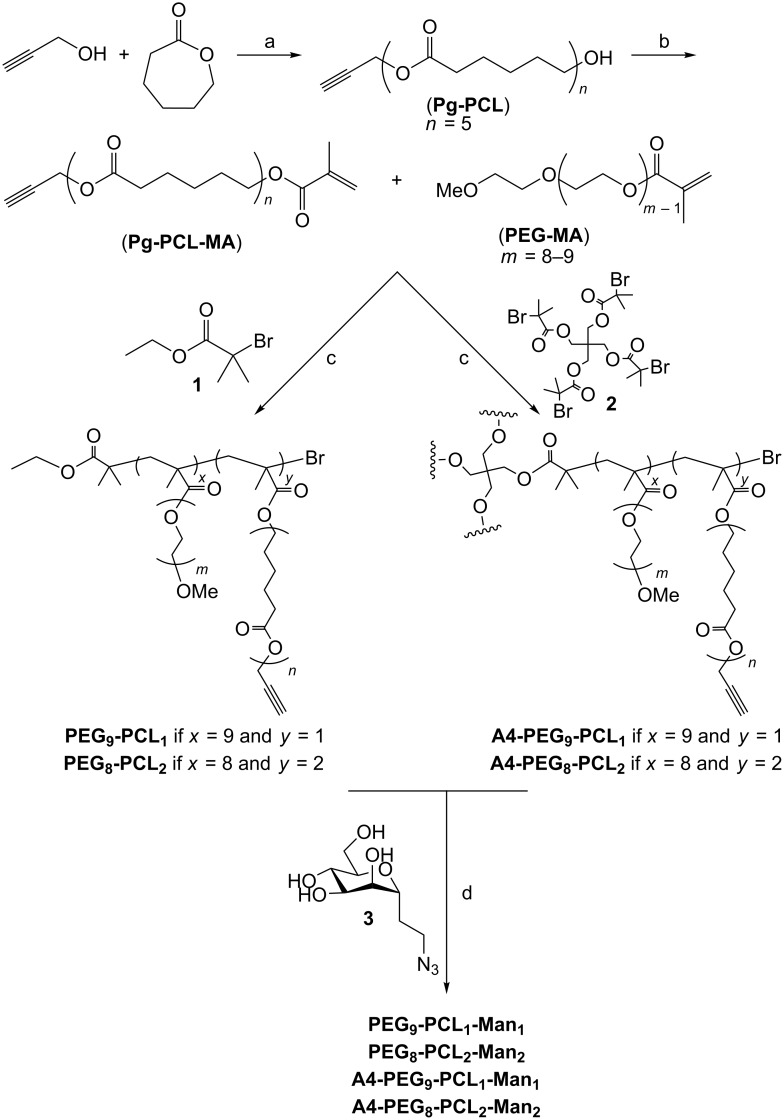
Synthetic pathway to obtain linear and four-arm mannosylated copolymers.

Propargyl-poly(ε-caprolactone)-methacrylate (**Pg-PCL-MA**) was prepared in two steps starting from ε-caprolactone, through a bulk ROP initiated by propargyl alcohol and catalyzed by tin(II) 2-ethylhexanoate [25] (Scheme 1, step a), targeting a degree of polymerization (DP) equal to 5. Reaction conversions were monitored by means of ^1^H NMR analysis; almost full monomer conversion (99%) was obtained after 6 h. The dispersity of the purified polymer was 1.19. Subsequent methacrylation of the terminal hydroxy group was accomplished in high conversions (≥95%) using freshly distilled methacryloyl chloride, in order to remove cyclic reactive impurities [26] (Scheme 1, step b).

### Synthesis of the clickable alkyne polymers **(A4-)PEG*_x_*-PCL*_y_***

Polymeric backbones with the general formula **(A4-)PEG*_x_*-PCL*_y_*** were synthesized through ATRP, randomly copolymerizing different percentages of commercially available poly(ethylene glycol)methyl ether methacrylate (**PEG-MA**, average *M*_n_ = 500 Da, with 8 or 9 ethylene oxide repeating units) and propargyl-poly(ε-caprolactone)-methacrylate (**Pg-PCL-MA**) in the presence of Cu(I)Br/1,1,4,7,10,10-hexamethyltriethylenetetramine (HMTETA) catalyst [27]. Ethyl α-bromoisobutyrate (**1**) and pentaerythritol tetrakis(2-bromoisobutyrate) (**2**) were chosen as the initiators for linear and four-arm polymers, respectively (Scheme 1, step c).

**Figure 2:**
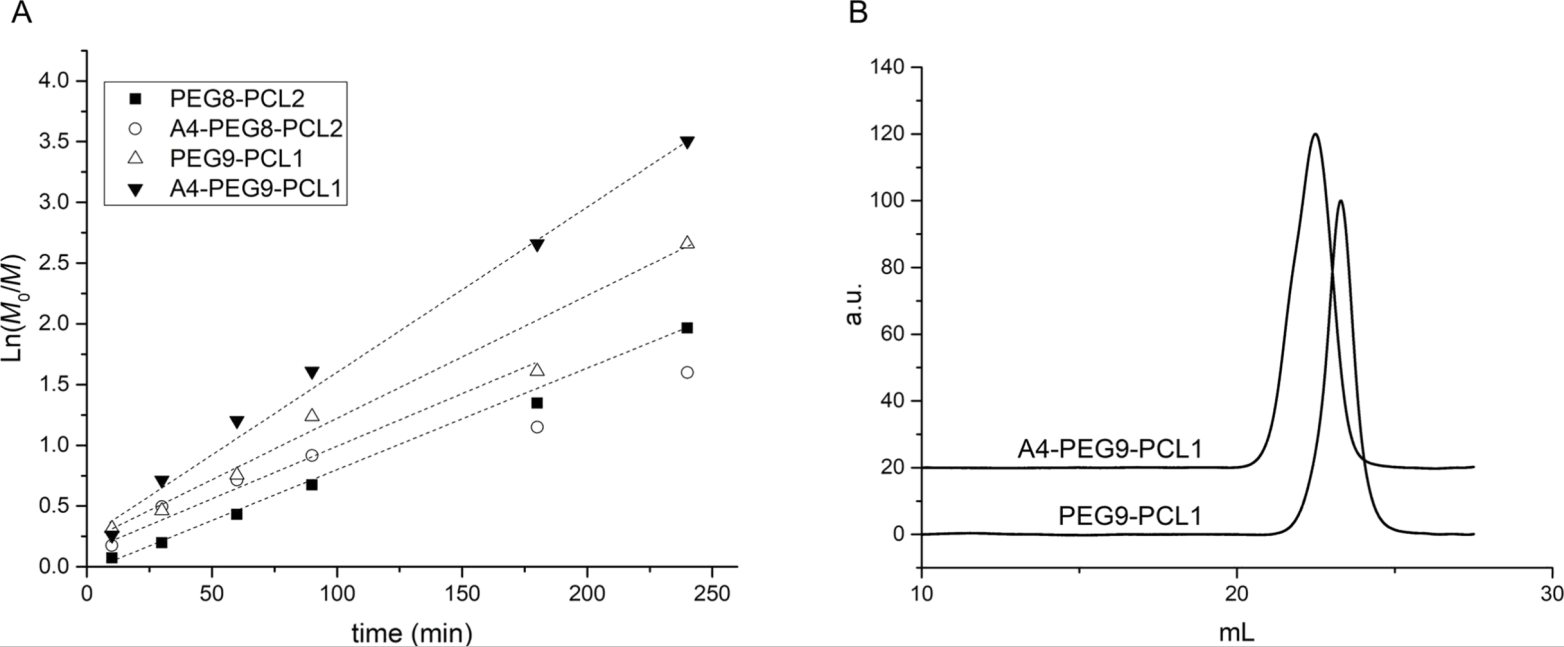
A) Kinetic profiles obtained for the synthesis of **(A4-)PEG_9_-PCL_1_** and **(A4-)PEG_8_-PCL_2_** copolymers. Reactions were conducted using CuBr/HMTETA as catalytic system, at 50 °C under nitrogen atmosphere. B) GPC chromatograms of **PEG_9_-PCL_1_** and **A4-PEG_9_-PCL_1_**.

A DP value of 10 (for the linear polymers and for each branch of the four-arm ones) was targeted, in order to obtain macromolecules of different valencies, while maintaining relatively short and flexible polymer chains, which minimize steric hindrance during lectin binding. Different percentages of PEG and PCL macromonomers were used, since the number of PCL side chains defined the final number of pendant mannose, as well as the self-assembly behavior in water. Remarkably, when the ratios PEG/PCL were 9:1 or 8:2, the polymerizations achieved high conversions (≥87% after 6 h), as calculated from ^1^H NMR spectroscopy results (see Figure S2, Supporting Information File 1). The semilogarithmic kinetic plots revealed first-order kinetics (Figure 2A), demonstrating a good reaction control over the polymer molecular weight and the molecular weight distribution, which was also confirmed by dispersity Ð values lower than 1.24 (Figure 2B, Table 1). The deviation of the number average molecular weight calculated by ^1^H NMR from the value obtained by GPC (which was substantial for the four-arm polymers) was likely to be caused by the polystyrene calibration and by the intrinsic limitation of the GPC to determine the exact molecular weight of comb-like and hyperbranched polymers, as observed in numerous earlier publications [28].

**Table 1:** Summary of properties of the copolymers obtained by ATRP. PEG/PCL macromonomers molar ratio, conversion, number average molecular weight obtained by ^1^H NMR (*M*_n,NMR_) and GPC (*M_n_*_,GPC_), and dispersity Ð (by GPC) are listed in the table.

polymer	PEG/PCL [mol/mol]	conversion (%)	*M*_n,NMR_ [g/mol]	*M*_n,GPC_ [g/mol]	Ð

**PEG_9_-PCL_1_**	9/1	95	5040	6090	1.24
**PEG_8_-PCL_2_**	8/2	91	4990	6301	1.06
**PEG_7_-PCL_3_**	7/3	58	3425	—	—
**A4-PEG_9_-PCL_1_**	9/1	98	20780	9640	1.11
**A4-PEG_8_-PCL_2_**	8/2	87	18760	11340	1.06

Increasing the percentage of PCL to PEG/PCL = 7:3, the reactions stopped at a conversion of around 58% (Table 1 and Figure S3, Supporting Information File 1). It is known that copper-catalyzed ATRP can be complicated by the presence of monomers bearing triple bonds, which can indeed coordinate the copper species. Moreover, also the acetylene group can be subjected to radical addition and subsequent polymerization and cross-linking [29]. For this reason, polymerizations using PCL monomers bearing protected triple bonds were also performed [30]. However, no improvements were obtained even by using trimethylsilyl-protected-Pg-PCL-MA, the homopolymerization of which achieved a conversion of 55% (see Supporting Information File 1), comparable to the homopolymerization of Pg-PCL-MA (43%). The use of a more active catalytic system such as *N*,*N*,*N*′,*N*′′,*N*′′-pentamethyldiethylenetriamine (PMDETA) [31] did not improve the outcome of the reaction. The characterisation of the copolymer with a PEG/PCL ratio of 7:3 was therefore abandoned, and further study was focused on the copolymers with higher PEG/PCL ratios. The copolymerization of **PEG-MA** and **TMS-Pg-PCL-MA** in an 8:2 ratio catalyzed by CuBr/PMDETA achieved almost full conversions, but the kinetic was not linear (Figure S10, Supporting Information File 1), whilst the analogous reaction with a **PEG-MA**/**Pg-PCL-MA** ratio of 8:2 stopped at a conversion value of 65%.

Final products were purified through filtration over a neutral alumina pad to remove the copper catalyst, followed by precipitation in diethyl ether.

### Synthesis of the mannosylated polymers

**Figure 3:**
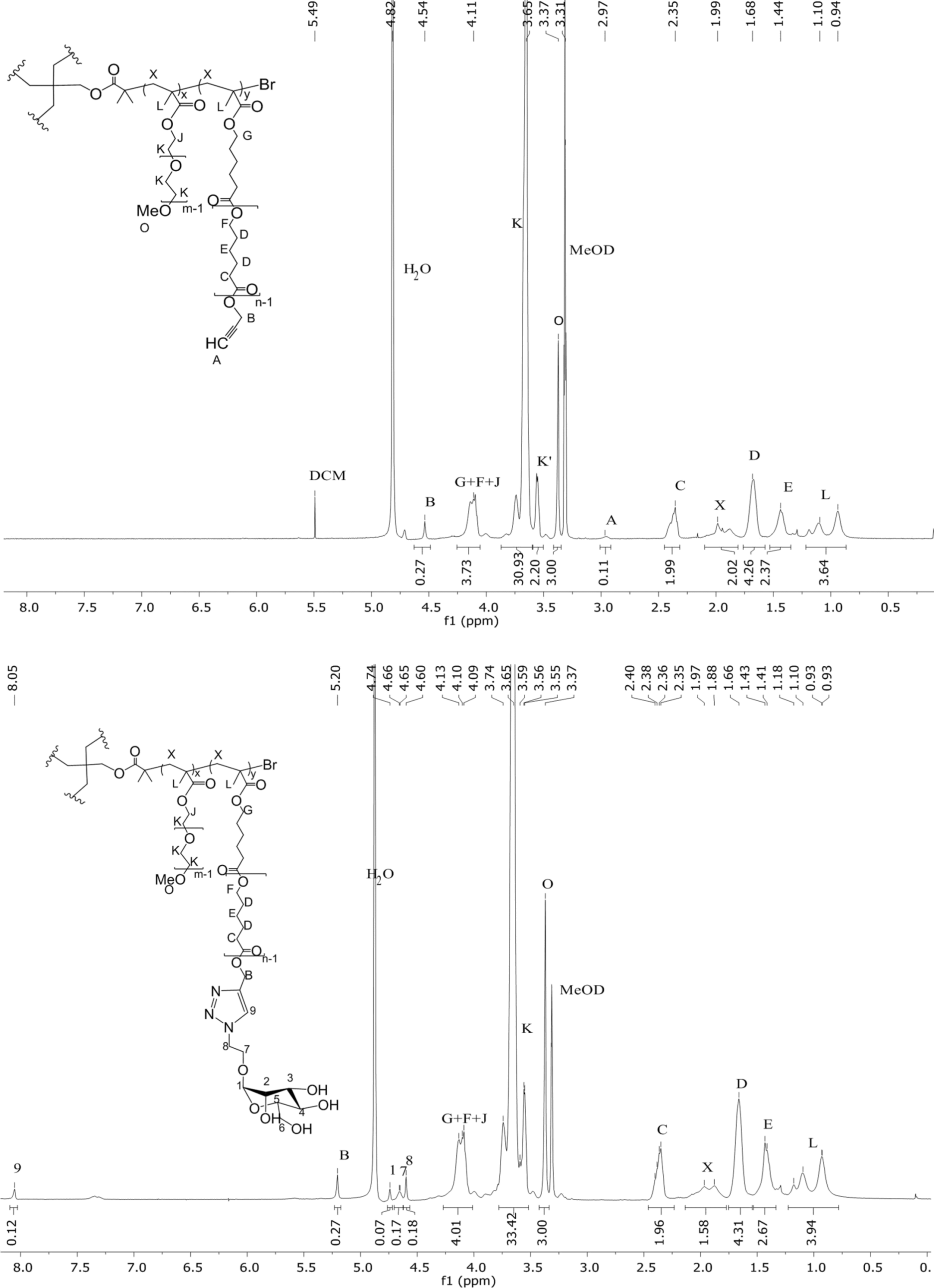
Comparison of the ^1^H NMR spectrum of one selected polymer (**A4-PEG_8_-PCL_2_**, MeOD) before (upper panel) and after (lower panel) the mannosylation reaction. The disappearance of the peak of the alkyne proton (A, upper) and the appearance of the peak of the triazole (9, lower), together with the presence of the Man-H1 proton (1, lower) in the product demonstrate the formation of the mannosylated polymer (**A4-PEG_8_-PCL_2_-Man_2_**).

The sugar functionalization of the polymeric backbone was performed through CuAAC click reaction (Scheme 1, step d), by combining the triple bonds with 2-azidoethyl α-ᴅ-mannopyranoside (**3**), synthesized as already reported [32] (see Supporting Information File 1, Figure S1).The reaction conditions for the CuAAC were chosen according to [33]. In particular, the catalytic copper species was produced in situ by reducing copper(II) sulfate pentahydrate with sodium ascorbate, in the presence of tris[(1-benzyl-1*H*-1,2,3-triazol-4-yl)methyl]amine (TBTA) as copper(I)-stabilizing ligand. Both the actual PCL percentage and the molecular weight of the reacting polymers were calculated according to ^1^H NMR analysis. An excess of 1.5 equiv of azide-bearing mannose **3** with respect to each triple bond was used. 2-azidoethyl α-ᴅ-mannopyranoside consumption was assessed through thin layer chromatography (TLC), whilst the triple bond conversion was monitored by means of ^1^H NMR analysis. The disappearance of the alkyne proton peak (3.0 ppm) and the appearance of a signal for the C=C–H proton of triazole (8.1 ppm) were observed (Figure 3). The shift of the peak corresponding to the methylene group near the triple bond (4.5 ppm) and the appearance of the signal of mannose anomeric protons (Man-H1, 4.7 ppm) in the product spectrum also confirmed the functionalization.

Reactions were completed after three days. The crudes were purified by dialysis against water, and the final products **(A4-)PEG*_x_*-PCL*_y_*-Man*_y_*** were lyophilized, achieving moderate to high yields.

### Nanoparticle formation and size characterization

**Figure 4:**
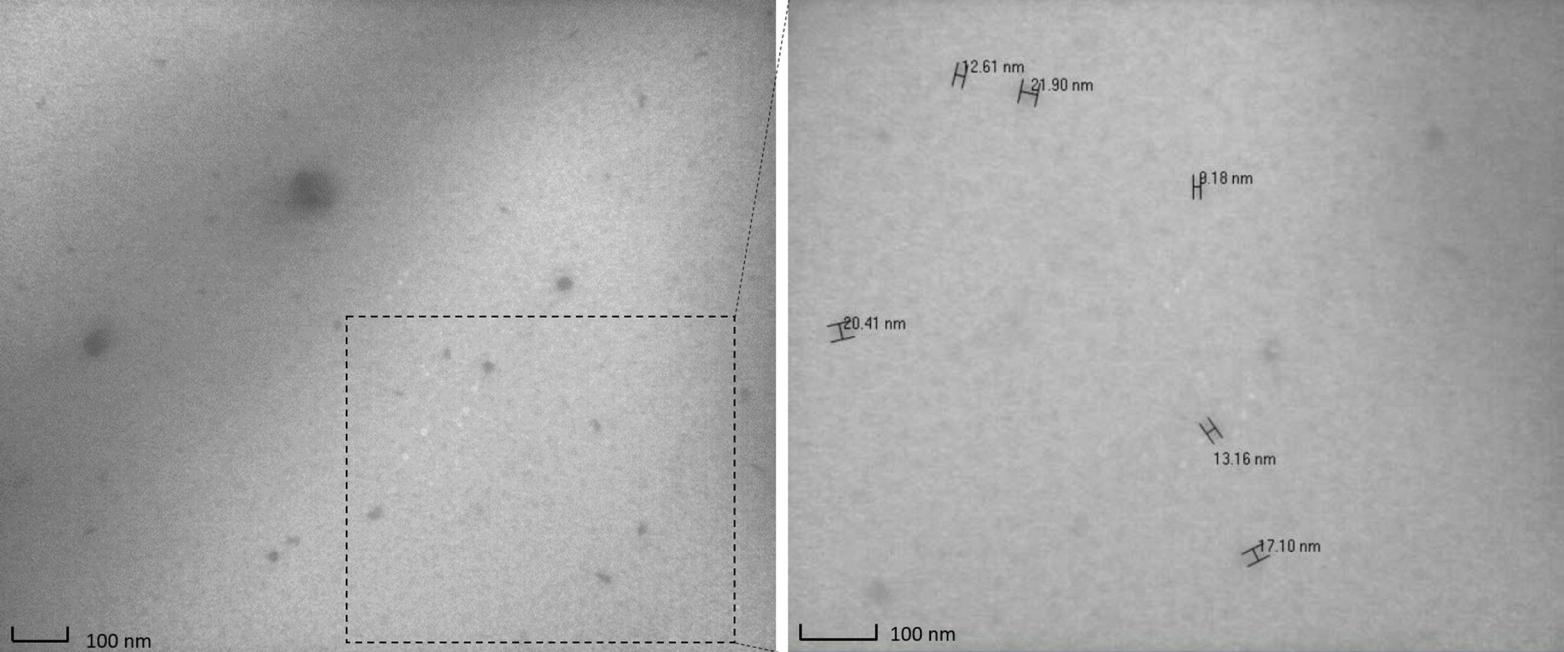
TEM images of **A4-PEG_8_-PCL_2_** 10 mg/mL in water; size distribution is dominated by small nanoparticles (diameter ≤ 20 nm).

Dynamic light scattering (DLS) was used to determine the hydrodynamic diameter (*D*_h_) of the polymers and their glyco-derivatives, once dispersed in aqueous solution. Firstly, the non-mannosylated polymers were tested in three different media (H_2_O, 10 mM PBS, 0.9% w/v NaCl) and at three different temperatures (10, 25 and 37 °C; concentration of 1 mg/mL, see Supporting Information File 1, Figures S11–S22, Tables S1–S4). In any medium, polymers formed nanoparticles with *D*_h_ varying from 7.5 to 11.3 nm, a size range which corresponds to 99–100% of the volume distribution. The remaining 0–1% was due to the presence of aggregates with a size of hundreds of nanometers as revealed by the scattering intensity distributions (Supporting Information File 1). Because of the limitation of the CONTIN algorithm used by this DLS analysis to determine a bimodal distribution of particle sizes [34], the predominance of small nanoparticles was confirmed by transmission electron microscopy (TEM) imaging (Figure 4), where dehydrated flattened particles of small size (10–20 nm) are shown together with few larger particles (50–100 nm).

Comparing the results obtained at the three different temperatures, it was observed that these polymers were not thermoresponsive, although PEG-copolymers may generally present a lower critical solubility temperature (Tables S1–S4) [35]. For our purposes, this could be an advantage, since they maintain the same size under different operation conditions: storage, room and body temperature.

**Figure 5:**
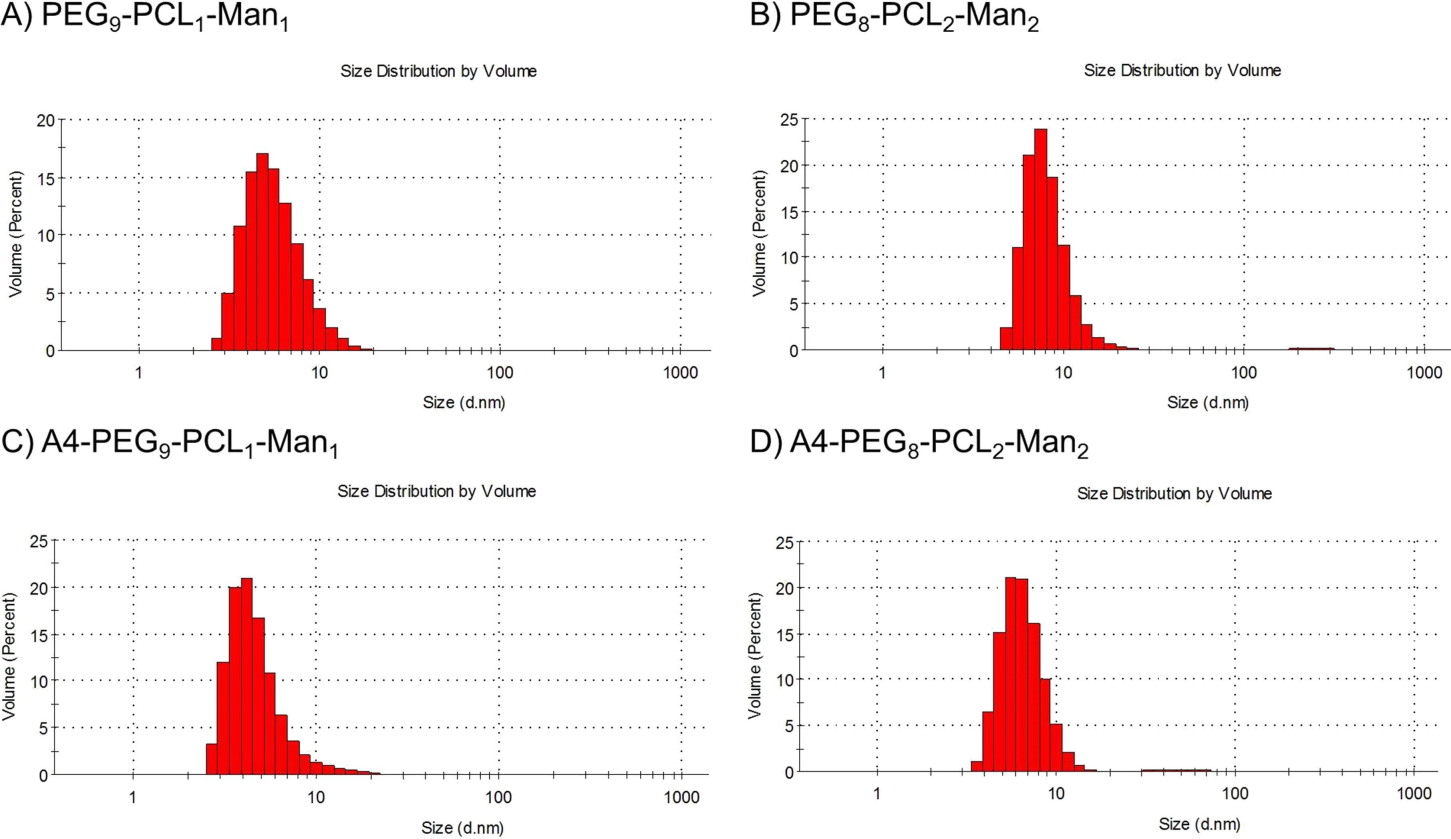
DLS size distribution (vol %) of the glycopolymers **PEG_9_-PCL_1_-Man_1_** (A), **PEG_8_-PCL_2_-Man_2_** (B), **A4-PEG_9_-PCL_1_-Man_1_** (C), **A4-PEG_8_-PCL_2_-Man_2_** (D), tested in water (1 mg/mL) at 25 °C.

Also the DLS distributions of the mannosylated **(A4-)PEG*_x_*-PCL*_y_*-Man*_y_*** glycopolymers in aqueous media (water and HBS buffer) were characterized by two populations. The volume distribution was still dominated by small-size nanomaterials (*D*_h_ ≈ 10 nm, Figure 5), although their intensity generally decreased with respect to larger colloids (Table 2 and Supporting Information File 1, Figures S23 and S24). This tendency may be due to the presence of the mannose residues that, being connected to the hydrophobic PCL chains, could affect the self-assembly of the macromolecules and generate some aggregates. Remarkably, four-arm species had a slightly lower *D*_h_ than the corresponding linear species.

**Table 2:** DLS analyses of polymers and corresponding glycopolymers in water (1 mg/mL) at 25 °C.

general formula	valency	hydrodynamic radius (nm) (intensity (%))	
		polymer	glyco derivative

**PEG_9_-PCL_1_-(Man_1_)**	1	8.5 (73)	8.6 (83)
**PEG_8_-PCL_2_-(Man_2_)**	2	10.2 (73)	10.8 (6)
**A4-PEG_1_-PCL_1_-(Man_1_)**	4	8.1 (79)	5.8 (12)
**A4-PEG_8_-PCL_2_-(Man_2_)**	8	9.6 (63)	8.0 (10)

### Turbidity assay

**Figure 6:**
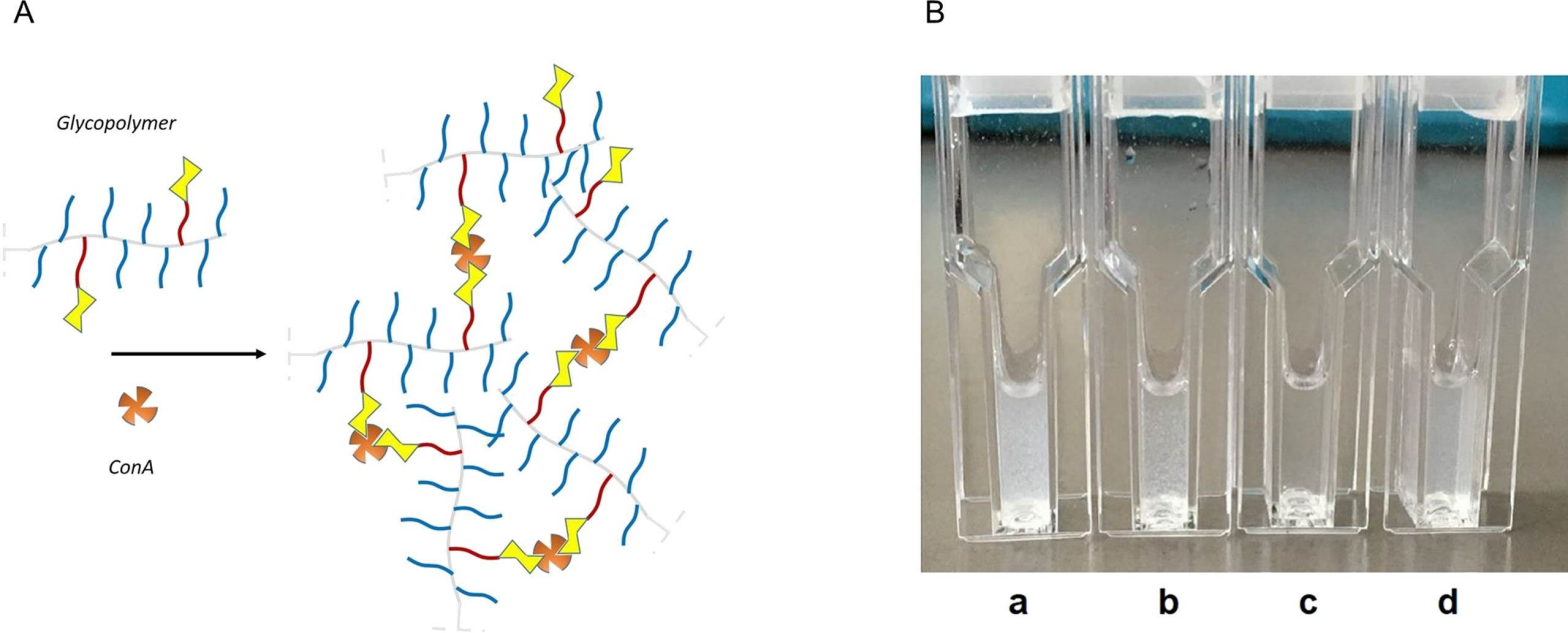
A) Schematic representation of Con A clustering by multivalent ligands. B) Con A suspensions after interaction with (a) **PEG_8_-PCL_2_-Man_2_** (turbid), (b) **A4-PEG_8_-PCL_2_-Man_2_** (turbid), (c) **PEG_9_-PCL_1_-Man_1_** (transparent) and (d) **A4-PEG_9_-PCL_1_-Man_1_** (turbid).

**Figure 7:**
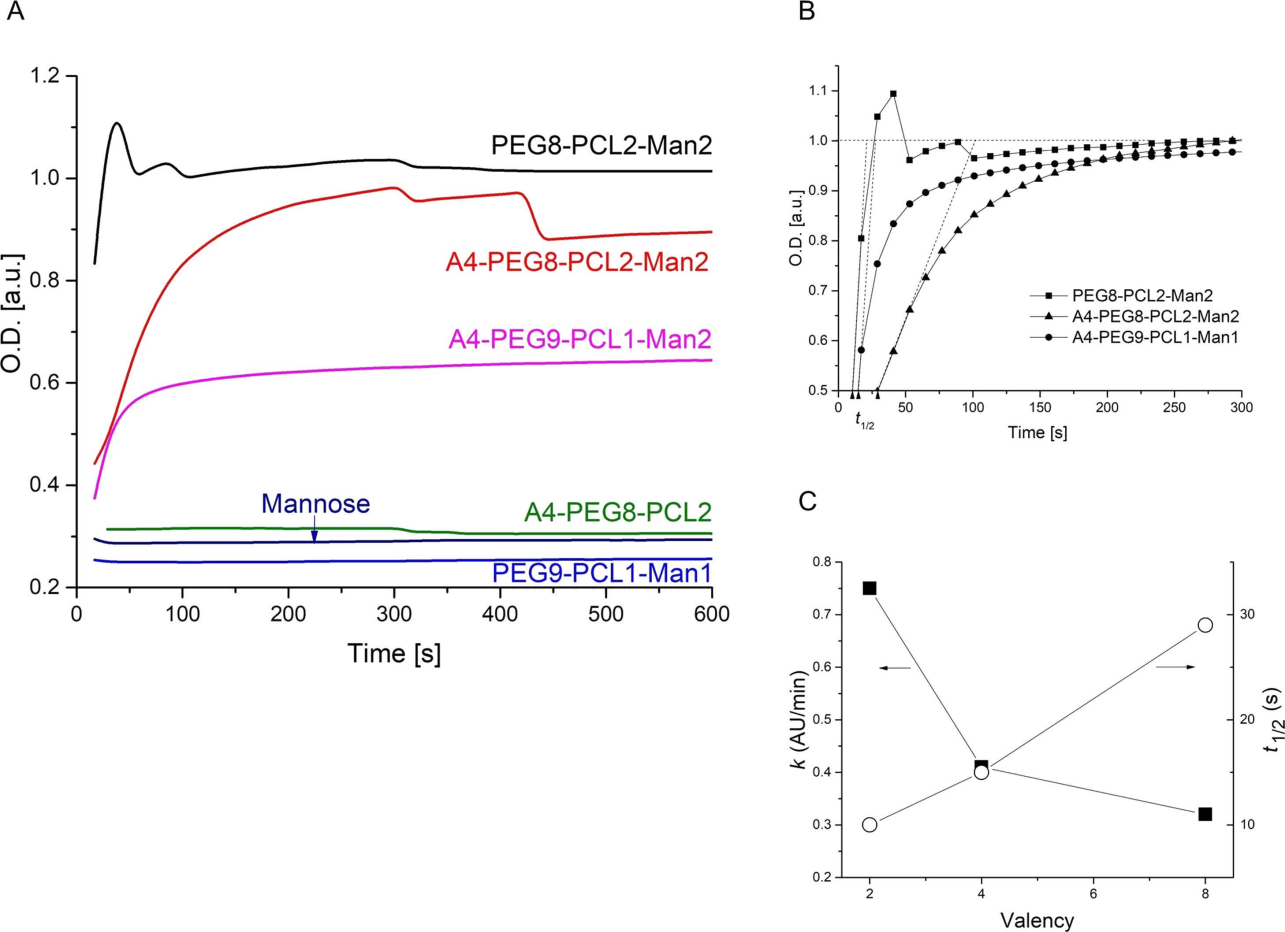
Turbidimetric assay results. A) Optical density (OD) data were recorded at 420 nm every 12 s for 10 min. B) *t*_1/2_ was calculated as the time to reach half (OD= 0.5 a.u.) of the maximum optical density in normalized OD curve. The initial slope of the OD curve was used to determine the initial aggregation rate *k*. C) *k* (AU/min) and *t*_1/2_ plotted as function of mannose valency.

The ability of glycopolymers to bind lectins was assessed using Concanavalin A (Con A), a model lectin able to recognize α-ᴅ-mannosyl and β-ᴅ-glucosyl residues. At physiological pH values, Con A is a tetramer, composed by four 26 kDa monomeric units, each of them possessing one coordination site [36–37]. When multivalent carbohydrate compounds bridge multiple Con A tetramers, insoluble clusters are formed, turning the originally transparent solution turbid (Figure 6B). Each single macromolecule may interact with many Con A receptors, although steric effects prevent binding of every mannose residue [38] (Figure 6A). Glycopolymers were tested in the presence of Con A in HBS buffer at pH 7.4. A fixed mannose concentration 2.5 times higher than the Con A binding site concentration was used, and turbidity was measured using an UV–vis spectrometer (Figure 7). The kinetics of glycopolymer-induced clustering were also investigated. In particular, the initial rates of Con A clustering, expressed as arbitrary units per minute (*k*, AU/min), were derived from the initial slope of the curves; the time to reach half of the maximum turbidity (*t*_1/2_) was determined from the endpoint of precipitation (Table 3).

**Table 3:** Initial rates of Con A precipitation (*k*, AU/min) and time to reach half of the maximum turbidity (*t*_1/2_, s) in turbidity assays.

Glycopolymer	Valency	*k* (AU/min)	*t*_1/2_ (s)

**PEG_9_-PCL_1_-Man_1_**	1	—	—
**PEG_8_-PCL_2_-Man_2_**	2	0.75	10
**A4-PEG_9_-PCL_1_-Man_1_**	4	0.41	15
**A4-PEG_8_-PCL_2_-Man_2_**	8	0.32	29

The curves of the optical density (OD) measured as a function of the time (Figure 7) confirmed that polymers without mannose residues (**A4-PEG_8_-PCL_2_**) and mannose itself were not able to induce Con A clustering. In contrast, **PEG_8_-PCL_2_-Man_2_**, **A4-PEG_8_-PCL_2_-Man_2_** and **A4-PEG_9_-PCL_1_-Man_1_** were able to bind Con A in a multivalent fashion, leading to an increase of turbidity. Interestingly, turbidity was not observed with the monovalent compound **PEG_9_-PCL_1_-Man_1_**, suggesting that the polymer and the resulting nanosized colloid in water do not have a suitable valency for the formation of Con A clusters. We assumed that the mannose residues were not hidden within the polymer colloids but were exposed for binding, since all the other glycopolymers did precipitate the lectin. The divalent compound **PEG_8_-PCL_2_-Man_2_** was the most efficient agglutinating agent of the series, even more potent than the octa- and tetra-valent polymers. In the case of the divalent compound **PEG_8_-PCL_2_-Man_2_**, the absorbance quickly reached a plateau and then remained almost constant, although a stepwise variation of the OD indicated a partial precipitation of the aggregates, as shown in Figure 6B. The same happened for the octavalent glycopolymer **A4-PEG_8_-PCL_2_-Man_2_**, but with a much lower initial rate. This behavior was already reported in the literature and it was ascribed to the slower Con A precipitation and the formation of cross-linked complexes over time [12,38–39]. Regarding the four-arm macromolecules, the tetravalent **A4-PEG_9_-PCL_1_-Man_1_** showed a similar behavior as **A4-PEG_8_-PCL_2_-Man_2_**, but it reached a much lower plateau, which indicated the formation of smaller colloidal clusters when the valency was reduced from 8 to 4. Moreover, the octavalent compound seemed to bound Con A more slowly (*k*-octa = 0.32 AU/min vs *k*-tetra = 0.41 AU/min, Table 3). This result may be ascribed to an effect of the different polymer composition on the aggregation kinetics. As general trend, the rate constant *k* of Con A clustering decreased as the valency increased, and consequently the time to reach half of the maximum turbidity *t*_1/2_ increased as the valency increased (Table 3 and Figure 7C).

According to these results, it seemed that the Con A clustering activity of these macromolecules depended not only on their valency, but also on their molecular weight and architecture [40]. For example, the best result obtained for the divalent polymer, which has a lower mannose density, may depend on its lower steric hindrance, which results in a more efficient Con A binding.

## Conclusion

A series of amphiphilic polymers, with linear or four-arm structure and consisting of a random sequence of PEG and PCL blocks, was successfully synthesized via a combination of ROP and ATRP. Polymers were subsequently mannosylated through a CuAAC click reaction, obtaining final glycopolymers with a mannose valency of 1, 2, 4 or 8. This synthetic strategy was exploited to produce well-defined nanomaterials that self-assemble in aqueous media forming ultrasmall nanoparticles. The ability of these glyco-functionalized nanoparticles to efficiently expose mannose to lectins was investigated with the model Concanavalin A, through turbidity assays. While the monovalent macromolecule was not able to induce Con A clustering, the linear divalent glycopolymer had a binding kinetics faster than the other four-arm compounds, and tetravalent one was even more active than the octavalent. These results indicated that besides mannose valency, macromolecular architecture can deeply influence the capability to bind lectins. According to their tunable characteristics, these glycopolymers can be potentially utilized as targeted anti-pathogenic nanomaterials and as well as for cell-targeted drug delivery.

## Experimental

### General procedures

Chemicals were purchased by commercial sources and used without further purification, unless otherwise indicated. Polyethylene glycol methyl ether (*M*_n_ = 500 Da) was purchased from Sigma-Aldrich. 2-Azidoethyl α-ᴅ-mannopyranoside (**3**) was synthesized as already described [32]. When anhydrous and oxygen-free conditions were required, the reactions were performed under nitrogen atmosphere. Inhibitor-free THF used for ATRP reactions was degassed prior use; THF used for click reaction was dried over Na/benzophenone and freshly distilled. Thin-layer chromatography (TLC) was performed on Silica Gel 60 F254 plates (Merck) with UV detection (254 and 365 nm) or using appropriate developing solutions. Flash column chromatography was performed on silica gel 230–400 mesh (Merck). Automated flash chromatography was performed on a Biotage® Isolera™ Prime system. NMR experiments were recorded on a Bruker AVANCE 400 MHz instrument at 298 K. Chemical shifts (δ) are reported in ppm downfield from the deuterated solvent as internal standard, coupling constants (*J*) in Hz. The ^1^H NMR resonances of compounds were assigned with the assistance of COSY and HSQC experiments. HSQC experiments were also used to assign the chemical shift of protons overlapping with the solvent signals. Signals were abbreviated as s, singlet; bs, broad singlet; d, doublet; t, triplet; q, quartet; m, multiplet. Sugar protons were numbered as customary. GPC analyses were carried out with Jasco instrument and following set up: 2055i auto sampler; RI-2031 refractive index detector; CO-2060 plus oven column; PU-2080 pump; three PLgel 300 mm∙7.5 mm (5 μm particle size) (10E4, 10E5, 500 A) and a PLgel 50 mm∙7.5 mm (5 μm particle size) guard, using THF as eluent at 35 °C. The system was controlled using polystyrene calibration kits (by RESTEK and Sigma-Fluka); samples were dissolved in THF at a concentration of 4 mg/mL and filtered (PTFE filters, 0.45 µm).

**Synthesis of propargyl-poly(**ε**-caprolactone) (Pg-PCL)**. **Pg-PCL** was synthesized as reported in a previous work [25]. Briefly, ε-caprolactone (20 g, 0.175 mol, 5 equiv) was added in a round flask and heated up to 130 °C. A second yellowish solution containing propargyl alcohol (2.04 mL, 0.035 mol, 1 equiv) and tin(II) 2-ethylhexanoate (Sn(Oct)_2_, 0.0709 g, 0.175 mmol, 0.005 equiv) was prepared, stirred under a nitrogen flow for 20 min, and then added to the ε-caprolactone. The reaction mixture was stirred for 6 h at 130 °C, then stopped by cooling the flask to room temperature. Purification was carried out by dissolving the crude in 22 mL of methanol and dropping it in 1320 mL of vigorously stirred water. The resulting precipitate was isolated by removing the supernatant, dissolved in CH_2_Cl_2_ and anhydrified with anhydrous sodium sulphate. Subsequently it was filtered, and the filtrate was dried under reduced pressure, obtaining 20 g of final product **Pg-PCL** as a colorless viscous oil. Conversion_ε-CL_ = 99%; conversion_propargyl alcohol_ = 96%; yield = 92%; *M*_n,NMR_ = 716 g·mol^−1^; *M*_n,GPC_ = 1345 g·mol^−1^; PDI = 1.19 (relative to linear polystyrene). ^1^H NMR (400 MHz, CDCl_3_) δ 4.65 (d, *J* = 2.4 Hz, 2H, -C*H*_2_-CCH), 4.03 (t, *J* = 6.6 Hz, 2H·(*n* − 1), -C*H*_2_-OC(O)-), 3.61 (t, *J* = 6.5 Hz, 2H, -C*H*_2_-OH), 2.45 (t, *J* = 2.4 Hz, 1H, -CH_2_-CC*H*), 2.38 – 2.19 (m, 2H·*n*, -OC(O)-C*H*_2_-), 1.70–1.52 (m, 4H·*n*, -OC(O)-CH_2_C*H*_2_-, -C*H*_2_CH_2_-OC(O)-), 1.45–1.24 (m, 2H·*n*, -CH_2_C*H*_2_CH_2_-).

**Synthesis of propargyl-poly(ε-caprolactone)-methacrylate (Pg-PCL-MA)**. **Pg-PCL-MA** was synthesized as reported in a previous work [25]. Briefly, **Pg-PCL** (5 g, 0.007 mol, 1 equiv) was added in a round flask and three cycles of vacuum–nitrogen were performed. 167 mL of dry toluene were added under nitrogen flow and the flask was cooled to 0 °C for 30 min. Triethylamine (1.46 mL, 0.01 mol, 1.5 equiv) and freshly distilled methacryloyl chloride (1.10 mL, 0.01 mol, 1.5 equiv) were sequentially added to the reaction mixture. The reaction mixture was stirred at room temperature under dynamic nitrogen atmosphere for 15 min and under static nitrogen atmosphere overnight. The crude was filtered through a celite pad (*h* = 6 cm, Φ = 2 cm), washing with toluene, and then the filtrate was evaporated under reduced pressure. The residue was taken up in 112.5 mL of CH_2_Cl_2_, washed with brine (3 × 45 mL) and dried over anhydrous sodium sulfate. The solvent was evaporated under vacuum, obtaining 4.14 g of final product **Pg-PCL-MA** as a pale yellow viscous oil. Conversion_OH->OMA_ = 95%; yield = 75%; *M*_n,NMR_ = 740 g·mol^−1^; *M*_n,GPC_ = 1380 g·mol^−1^; PDI = 1.05 (relative to linear polystyrene). ^1^H NMR (400 MHz, CDCl_3_) δ 6.04 (s, 1H, *H*_2_C-C(CH_3_)-), 5.50 (s, 1H, *H*_2_C-C(CH_3_)-), 4.63 (d, *J* = 2.4 Hz, 2H, -C*H*_2_-CCH), 4.10 (t, *J* = 13.1 Hz, 2H, -C*H*_2_-O-MA), 4.02 (t, *J* = 6.7 Hz, 2H·(*n* − 1), -C*H*_2_-OC(O)-), 2.44 (t, *J* = 2.4 Hz, 1H, -CH_2_-CC*H*), 2.38–2.19 (m, 2H·*n*, -OC(O)-C*H*_2_-), 1.89 (s, 3H, H_2_C-C(C*H*_3_)-), 1.75–1.50 (m, 4H·*n*, -OC(O)-CH_2_C*H*_2_-, -C*H*_2_CH_2_-OC(O)-), 1.50–1.25 (m, 2H·*n*, -CH_2_C*H*_2_CH_2_-).

**General procedure (1) for the synthesis of PEG*_x_*-PCL*_y_***. THF (inhibitor-free) was degassed under nitrogen for 10 min. **Pg-PCL-MA** (*y* equiv) and poly(ethylene glycol) methyl ether methacrylate (*M*_n_ = 500 Da, *x* equiv) were added in a Schlenk tube and three cycles of vacuum–nitrogen were performed. The catalyst solution was prepared as follows: Copper(I)bromide (500 mg) was inserted in a Schlenk tube and three cycles of vacuum–nitrogen were performed. THF (6 mL) and the ligand 1,1,4,7,10,10-hexamethyltriethylenetetramine (HMTETA, 0.95 mL) were added, obtaining a light green mixture that was stirred at room temperature under N_2_ for 10 min. Finally, THF, the catalyst solution (containing CuBr/HMTETA 1 equiv with respect to the initiator) and the initiator (1 equiv, ethyl 2-bromo-2-methylpropionate (**1**)) were added to the monomers. The reaction mixture was stirred for 6 h at 50 °C under nitrogen atmosphere. The purification was performed by filtering the reaction mixture through a neutral alumina pad (*h* = 1.5 cm/*m*_crude_ (g), Φ = 2 cm), washing with CH_2_Cl_2_. Then the filtrate was dried under reduced pressure and the resulting crude was dissolved in the minimum amount of CH_2_Cl_2_ (ca. 1 mg/mL) and dripped in cold diethyl ether (CH_2_Cl_2_/Et_2_O = 1:60). The mixture was stored at −20 °C for 3 h and then the resulting precipitate was isolated by removing the supernatant.

**General procedure (2) for the synthesis of A4-PEG*_x_*-PCL*_y_***. THF (inhibitor-free) was degassed under nitrogen for 10 min. **Pg-PCL-MA** (4*y* equiv) and poly(ethylene glycol) methyl ether methacrylate (*M*_n_ = 500 Da, 4*x* equiv) were added in a Schlenk tube and three cycles of vacuum–nitrogen were performed. The catalyst solution was prepared as follows: Copper(I)bromide (500 mg) was inserted in a Schlenk tube and three cycles of vacuum–nitrogen were performed. THF (6 mL) and the ligand 1,1,4,7,10,10-hexamethyltriethylenetetramine (HMTETA, 0.95 mL) were added, obtaining a light green mixture that was stirred at room temperature under N_2_ for 10 min. Finally, THF, the catalyst solution (containing CuBr/HMTETA 4 equiv with respect to the initiator) and the initiator (1 equiv, pentaerythritol tetrakis(2-bromo-isobutyrate, **2**) 80 mg/mL in THF) were added to the monomers. The reaction mixture was stirred for 6 h at 50 °C under nitrogen atmosphere. The purification was performed by filtering the reaction mixture through a neutral alumina pad (*h* = 1.5 cm/*m*_crude_ (g), Φ = 2 cm), washing with CH_2_Cl_2_. Then the filtrate was dried under reduced pressure and the resulting crude was dissolved in the minimum amount of CH_2_Cl_2_ (ca. 1 mg/mL) and dripped in cold diethyl ether (CH_2_Cl_2_/Et_2_O = 1:60). The mixture was stored at −20 °C for 3 h and then the resulting precipitate was isolated by removing the supernatant.

**Synthesis of PEG_9_-PCL_1_**. The reaction between **Pg-PCL-MA** (245 mg, 0.306 mmol, 1 equiv) and poly(ethylene glycol) methyl ether methacrylate (1.38 g, 2.751 mmol, 9 equiv) was performed according to general procedure (1) in THF ([initiator] = 0.068 M), in the presence of CuBr (44 mg, 0.306 mmol, 1 equiv) and HMTETA (0.083 mL, 0.306 mmol, 1 equiv). Ethyl 2-bromo-2-methylpropionate **1** (45 µL, 0,348 mmol, 1 equiv) was the initiator. 713 mg of final product **PEG_9_-PCL_1_** were obtained. Conversion = 95%; real PEG = 92%; yield = 62%; *M*_n,NMR_ = 5040 g·mol^−1^; *M*_n,GPC_ = 6090 g·mol^−1^; PDI = 1.24 (relative to linear polystyrene). ^1^H NMR (400 MHz, CDCl_3_) δ 4.67 (d, *J* = 2.4 Hz, 2H·*y*, -C*H*_2_-CCH), 4.19–3.82 (m, 2H·*n*·*y +* 2H·*x*, PCL: -C*H*_2,PCL_-OC(O)-backbone, -C*H*_2_-OC(O)-, PEG: -C*H*_2,PEG_-OC(O)-backbone), 3.75–3.50 (m, (4H·(*m* − 1) + 2H)·*x*, PEG: -C*H*_2_C*H*_2_-), 3.37 (s, 3H·*x*, -OCH_3_), 2.47 (bs, 1H·*y*, -CH_2_-CC*H*), 2.40–2.25 (m, (2H·*n*)·*y*, PCL: -OC(O)-C*H*_2_-), 2.08–1.72 (m, 2H·(*y* + *x*), -C*H*_2,backbone_-), 1.73–1.53 (m, (4H·*n*)·*y*, PCL: -OC(O)-CH_2_C*H*_2_-, -C*H*_2_CH_2_-OC(O)-), 1.43–1.27 (m, (2H·*n*)·*y*, PCL: -CH_2_C*H*_2_CH_2_-), 1.17–0.69 (m, 3H·(*y* + *x*), -C*H*_3,backbone_).

**Synthesis of PEG_8_-PCL_2_**. The reaction between **Pg-PCL-MA** (517 mg, 0.696 mmol, 2 equiv) and poly(ethylene glycol) methyl ether methacrylate (1.39 g, 2.784 mmol, 8 equiv) was performed according to general procedure (1) in THF ([initiator] = 0.068 M), in the presence of CuBr (50 mg, 0.348 mmol, 1 equiv) and HMTETA (0.095 mL, 0.348 mmol, 1 equiv). Ethyl 2-bromo-2-methylpropionate (**1**, 51 µL, 0.348 mmol, 1 equiv) was the initiator. 1.48 g of final product **PEG_8_-PCL_2_** were obtained. Conversion = 91%; real PEG = 82%; yield = 92%; *M*_n,NMR_ = 4990 g·mol^−1^; *M*_n,GPC_ = 6301 g·mol^−1^; PDI = 1.06 (relative to linear polystyrene). ^1^H NMR (400 MHz, CDCl_3_) δ 4.67 (d, *J* = 2.4 Hz, 2H·*y*, -C*H*_2_-CCH), 4.15–4.0 (m, (2H·*n*)·*y +* 2H·*x*, PCL: -C*H*_2,PCL_-OC(O)-backbone, -C*H*_2_-OC(O)-, PEG: -C*H*_2,PEG_-OC(O)-backbone), 3.75–3.51 (m, (4H·(*m* − 1) + 2H)·*x*, PEG: -C*H*_2_C*H*_2_-), 3.36 (s, 3H·*x*, -OCH_3_), 2.47 (bs, 1H·*y*, -CH_2_-CC*H*), 2.48–2.28 (m, (2H·*n*)·*y*, PCL: -OC(O)-C*H*_2_-), 1.95–1.70 (m, 2H·(*x* + *y*), -C*H*_2,backbone_-), 1.75–1.55 (m, (4H·*n*)·*y*, PCL: -OC(O)-CH_2_C*H*_2_-, -C*H*_2_CH_2_-OC(O)-), 1.45–1.30 (m, (2H·*n*)·*y*, PCL: -CH_2_C*H*_2_CH_2_-), 1.16–0.66 (m, 3H·(*x* + *y*), -C*H*_3,backbone_).

**Synthesis of A4-PEG_9_-PCL_1_**. The reaction between **Pg-PCL-MA** (240 mg, 0.3 mmol, 4 equiv) and poly(ethylene glycol) methyl ether methacrylate (1.35 g, 2.695 mmol, 36 equiv) was performed according to general procedure (2) in THF ([initiator] = 0.02 M), in the presence of CuBr (43 mg, 0.3 mmol, 4 equiv) and HMTETA (0.082 mL, 0.3 mmol, 4 equiv). Pentaerythritol tetrakis(2-bromo-isobutyrate) (**2**, 55 mg, 0.075 mmol, 1 equiv) was the initiator. 1.24 g of final product **A4-PEG_9_-PCL_1_** were obtained. Conversion = 98%; real PEG = 94%; yield = 85%; *M*_n,NMR_ = 20780 g·mol^−1^; *M*_n,GPC_ = 9640 g·mol^−1^; PDI = 1.11 (relative to linear polystyrene). ^1^H NMR (400 MHz, CDCl_3_) δ 4.66 (d, *J* = 2.5 Hz, 2H·*y*, -C*H*_2_-CCH), 4.20–3.95 (m, (2H·*n*)·*y +* 2H·*x*, PCL: -C*H*_2,PCL_-OC(O)-backbone, -C*H*_2_-OC(O)-, PEG: -C*H*_2,PEG_-OC(O)-backbone), 3.75–3.48 (m, (4H·(*m* − 1) + 2H)·*x*, PEG: -C*H*_2_C*H*_2_-),), 3.36 (s, 3H·*x*, -OCH_3_), 2.47 (bs, 1H·*y*, -CH_2_-CC*H*), 2.45–2.20 (m, (2H·*n*)·*y*, PCL: -OC(O)-C*H*_2_-), 2.05–1.80 (m, 2H·(*y* + *x*), -C*H*_2,backbone_-), 1.73–1.53 (m, (4H·*n*)·*y*, PCL: -OC(O)-CH_2_C*H*_2_-, -C*H*_2_CH_2_-OC(O)-), 1.48–1.30 (m, (2H·*n*)·*y*, PCL: -CH_2_C*H*_2_CH_2_-), 1.16–0.73 (m, 3H·(*y* + *x*), -C*H*_3,backbone_).

**Synthesis of A4-PEG_8_-PCL_2_**. The reaction between **Pg-PCL-MA** (660 mg, 0.89 mmol, 8 equiv) and poly(ethylene glycol) methyl ether methacrylate (1.78 g, 3.55 mmol, 32 equiv) was performed according to general procedure (2) in THF ([initiator] = 0.02 M), in the presence of CuBr (64 mg, 0.44 mmol, 4 equiv) and HMTETA (0.121 mL, 0.44 mmol, 4 equiv). Pentaerythritol tetrakis(2-bromo-isobutyrate) (**2**, 81 mg, 0.11 mmol, 1 equiv) was the initiator. 1.5 g of final product **A4-PEG_8_-PCL_2_** were obtained. Conversion = 87%; real PEG = 84%; yield = 74% *M*_n,NMR_ = 18760 g·mol^−1^; *M*_n,GPC_ = 11340 g·mol^−1^; PDI = 1.06 (relative to linear polystyrene). ^1^H NMR (400 MHz, CDCl_3_) δ 4.67 (d, *J* = 2.4 Hz, 2H·*y*, -C*H*_2_-CCH), 4.15–3.95 (m, (2H·*n*)·*y +* 2H·*x*, PCL: -C*H*_2,PCL_-OC(O)-backbone, -C*H*_2_-OC(O)-, PEG: -C*H*_2,PEG_-OC(O)-backbone), 3.75–3.51 (m, (4H·(*m* − 1) + 2H)·*x*, PEG: -C*H*_2_C*H*_2_-), 3.36 (s, 3H·*x*, -OCH_3_), 2.47 (bs, 1H·*y*, -CH_2_-CC*H*), 2.49–2.25 (m, (2H·*n*)·*y*, PCL: -OC(O)-C*H*_2_-), 2.0–1.75 (m, 2H·(*y* + *x*), -C*H*_2,backbone_-), 1.74–1.55 (m, (4H·*n*)·*y*, PCL: -OC(O)-CH_2_C*H*_2_-, -C*H*_2_CH_2_-OC(O)-), 1.45–1.30 (m, (2H·*n*)·*y*, PCL: -CH_2_C*H*_2_CH_2_-), 1.16–0.66 (m, 3H·(*y* + *x*), -C*H*_3,backbone_).

**General procedure for the CuAAC reaction**. In the optimized copper(I)‐catalyzed azide–alkyne cycloaddition (CuAAC) procedure, starting materials and reagents were added to the reaction mixture as solids or as solutions in water or THF. Water was degassed by bubbling with nitrogen, and THF was freshly distilled. The reagents were added to the reaction vessel in the following order: polymer (1 equiv, solid), TBTA (0.2 equiv, 27 mg/mL in THF), CuSO_4_·5H_2_O (0.1 equiv, 21 mg/mL in H_2_O), sodium ascorbate (0.4 equiv, 67 mg/mL in H_2_O) and, after 10 min, 2-azidoethyl α-ᴅ-mannopyranoside **3** (1.5 equiv per each triple bond, 32 mg/mL in H_2_O). The final concentration of the alkyne groups was 30 mM in a 1:1 THF/H_2_O mixture. The reaction mixture was stirred at room temperature for 3 days, under nitrogen atmosphere and protected from light, adding 0.25 equiv of sodium ascorbate after 2, 29 and 47 h. The sugar consumption was monitored by TLC (eluent: CHCl_3_/MeOH 7:3) and the product formation by ^1^H NMR. When intermediates were observed but the azide monovalent ligand **3** was totally consumed, the latter was added (0.75 equiv) together with additional 0.25 equiv of sodium ascorbate. The solvent was evaporated and the crude redissolved in water (9 mg/mL) and dialyzed against an excess (60 times) of water (regenerated cellulose membrane with MWCO = 3500 Da) for 1 day, changing the water three times. Final glycopolymers were lyophilized. Final ^1^H NMR spectra are reported in Figures S6–S9 (Supporting Information File 1).

**Synthesis of monovalent glycopolymer PEG_9_-PCL_1_-Man_1_**. The reaction between **PEG_9_-PCL_1_** (150 mg, 0.022 mmol_PCL_, 1 equiv_PCL_) and (2-azidoethyl)-α-ᴅ-mannopyranoside (**3**, 8.4 mg, 0.034 mmol, 1.5 equiv) was performed in 1:1 THF/H_2_O ([alkyne] = 30 mM) according to the general procedure. 124.1 mg of final product PEG_9_-PCL_1_-Man_1_ were obtained. Conversion = 100%; yield = 80%. ^1^H NMR (400 MHz, CD_3_OD) δ 8.04 (s, 1H·*y*, H_triazole_), 5.20 (s, 2H·*y*, -O-C*H*_2_-triazole), 4.74 (bs, 1H·*y*, H_1_^Man^), 4.72–4.62 (m, 4H·*y*, -N-C*H*_2_C*H*_2_-O-_linker_), 4.23–4.02 (m, (2H·*n*)·*y +* 2H·*x*, PCL: -C*H*_2,PCL_-OC(O)-backbone, -C*H*_2_-OC(O)-, PEG: -C*H*_2,PEG_-OC(O)-backbone), 3.74–3.52 (m, (2H + 4H·(*m* − 1))·*x*, PEG: -C*H*_2_C*H*_2_-), 3.37 (s, 3H·*x*, -OCH_3_), 2.45–2.30 (m, (2H·*n*)·*y*, PCL: -OC(O)-C*H*_2_-), 2.10–1.75 (m, 2H·(*y* + *x*), -C*H*_2,backbone_-), 1.74–1.55 (m, (4H·*n*)·*y*, PCL: -OC(O)-CH_2_C*H*_2_-, -C*H*_2_CH_2_-OC(O)-), 1.56–1.32 (m, (2H·*n*)·*y*, PCL: -CH_2_C*H*_2_CH_2_-), 1.23–0.76 (m, 3H·(*y* + *x*), -C*H*_3,backbone_).

**Synthesis of divalent glycopolymer PEG_8_-PCL_2_-Man_2_**. The reaction between **PEG_8_-PCL_2_** (150 mg, 0.05 mmol_PCL_, 1 equiv_PCL_) and (2-azidoethyl)-α-ᴅ-mannopyranoside (**3**, 19 mg, 0.075 mmol, 1.5 equiv) was performed in 1:1 THF/H_2_O ([alkyne] = 30 mM) according to the general procedure. 105 mg of final product **PEG_8_-PCL_2_-Man_2_** were obtained. Conversion = 100%; yield = 65%; ^1^H NMR (400 MHz, CD_3_OD) δ 8.05 (s, 1H·*y*, H_triazole_), 5.20 (s, 2H·*y*, -O-C*H*_2_-triazole), 4.74 (bs, 1H·*y*, H_1_^Man^), 4.69–4.52 (m, 4H·*y*, -N-C*H*_2_C*H*_2_-O-_linker_), 4.26–4.03 (m, (2H·n)·*y +* 2H·*x*, PCL: -C*H*_2,PCL_-OC(O)-backbone, -C*H*_2_-OC(O)-, PEG: -C*H*_2,PEG_-OC(O)-backbone), 3.80–3.50 (m, (2H + 4H·(*m* − 1))·*x*, PEG: -C*H*_2_C*H*_2_-), 3.37 (s, 3H·*x*, -OCH_3_), 2.48–2.28 (m, (2H·*n*)·*y*, PCL: -OC(O)-C*H*_2_-), 2.00–1.75 (m, 2H·(*x* + *y*), -C*H*_2,backbone_-), 1.75–1.50 (m, (4H·*n*)·*y*, PCL: -OC(O)-CH_2_C*H*_2_-, -C*H*_2_CH_2_-OC(O)-), 1.50–1.30 (m, (2H·*n*)·*y*, PCL: -CH_2_C*H*_2_CH_2_-), 1.24–0.80 (m, 3H·(*x* + *y*), -C*H*_3,backbone_).

**Synthesis of tetravalent glycopolymer A4-PEG_9_-PCL_1_-Man_1_**. The reaction between **A4-PEG_9_-PCL_1_** (150 mg, 0.017 mmol_PCL_, 1 equiv_PCL_) and (2-azidoethyl)-α-ᴅ-mannopyranoside (**3**, 6.3 mg, 0.025 mmol, 1.5 equiv) was performed in 1:1 THF/H_2_O ([alkyne] = 30 mM) according to the general procedure. 117.9 mg of final product **A4-PEG_9_-PCL_1_-Man_1_** were obtained. Conversion = 100%; yield = 76%. ^1^H NMR (400 MHz, CD_3_OD) δ 8.04 (s, 1H·*y*, H_triazole_), 5.20 (s, 2H·*y*, -O-C*H*_2_-triazole), 4.73 (bs, 1H·*y*, H_1_^Man^), 4.67–4.52 (m, 4H·*y*, -N-C*H*_2_C*H*_2_-O-_linker_), 4.22–4.05 (m, (2H·*n*)·*y +* 2H·*x*, PCL: -C*H*_2,PCL_-OC(O)-backbone, -C*H*_2_-OC(O)-, PEG: -C*H*_2,PEG_-OC(O)-backbone), 3.70–3.55 (m, (2H + 4H·(*m* − 1))·*x*, PEG: -C*H*_2_C*H*_2_-), 3.37 (s, 3H·*x*, -OCH_3_), 2.48–2.25 (m, (2H·*n*)·*y*, PCL: -OC(O)-C*H*_2_-), 2.05–1.80 (m, 2H·(*y* + *x*), -C*H*_2,backbone_-), 1.75–1.55 (m, (4H·*n*)·*y*, PCL: -OC(O)-CH_2_C*H*_2_-, -C*H*_2_CH_2_-OC(O)-), 1.50–1.30 (m, (2H·*n*)·*y*, PCL: -CH_2_C*H*_2_CH_2_-), 1.20–0.82 (m, 3H·(*y* + *x*), -C*H*_3,backbone_).

**Synthesis of octavalent glycopolymer A4-PEG_8_-PCL_2_-Man_2_**. The reaction between **A4-PEG_8_-PCL_2_** (150 mg, 0.044 mmol_PCL_, 1 equiv_PCL_) and (2-azidoethyl)-α-ᴅ-mannopyranoside (**3**, 16.6 mg, 0.067 mmol, 1.5 equiv) was performed in 1:1 THF/H_2_O ([alkyne] = 30 mM) according to the general procedure. 145.2 mg of final product **A4-PEG_8_-PCL_2_-Man_2_** were obtained. Conversion = 100%; yield = 90%. ^1^H NMR (400 MHz, CD_3_OD) δ 8.05 (s, 1H·*y*, H_Triazole_), 5.20 (s, 2H·*y*, -O-C*H*_2_-triazole), 4.74 (bs, 1H·*y*, H_1_^Man^), 4.70–4.58 (m, 4H·*y*, -N-C*H*_2_C*H*_2_-O-_linker_), 4.28–4.03 (m, (2H·*n*)·*y +* 2H·*x*, PCL: -C*H*_2,PCL_-OC(O)-backbone, -C*H*_2_-OC(O)-, PEG: -C*H*_2,PEG_-OC(O)-backbone), 3.74–3.55 (m, (2H + 4H·(*m* − 1))·*x*, PEG: -C*H*_2_C*H*_2_-), 3.37 (s, 3H·*x*, -OCH_3_), 2.48–2.30 (m, (2H·*n*)·*y*, PCL: -OC(O)-C*H*_2_-), 2.20–1.75 (m, 2H·(*y* + *x*), -C*H*_2,backbone_-), 1.73–1.60 (m, (4H·*n*)·*y*, PCL: -OC(O)-CH_2_C*H*_2_-, -C*H*_2_CH_2_-OC(O)-), 1.52–1.31 (m, (2H·*n*)·*y*, PCL: -CH_2_C*H*_2_CH_2_-), 1.25–0.77 (m, 3H·(*y* + *x*), -C*H*_3,backbone_).

### Particle size measurements by DLS

DLS analyses of polymers (1 mg/mL, filtered solutions with PTFE 0.45 µm filters) were performed using a Malvern Instrument Zetasizer Nano ZS instrument equipped with a 4 mW He–Ne laser operating at λ = 634 nm. Particle size distribution by scattering intensity (%) was determined by the CONTIN algorithm, as provided by the Zetasizer software (Malvern, UK). Particle size distribution by volume (%) was calculated from the scattering intensity distributions by the Zetasizer software, by setting the refractive index of the material R.I. = 1.465, which corresponds nearly to the refractive indices of poly(ethylene glycol) methacrylate *M*_n_ = 500 Da (R.I. = 1.467) as well as of the ε**-**caprolactone repeating units (R.I. = 1.463), as reported by the supplier (Sigma Aldrich).

### Transmission electron microscopy

TEM images were acquired with a DeLong America LVEM5 microscope, equipped with a field-emission gun and operating at 5 kV. TEM samples were prepared by dropping 10 µL of sample (10 mg/mL in water) on a copper grid (400 mesh) placed on filter paper. The grid was left to dry overnight for evaporating the solvent.

### Turbidimetric assay

This assay was performed in filtered (PTFE, 0.2 μm) HEPES-buffered saline (HBS), containing 10 mM HEPES, 150 mM NaCl and 1 mM CaCl_2_ at pH 7.4, according to a procedure already reported in the literature [38]. The stock solution of Con A (6 μM with respect to the tetramer, *M*_W_ = 104000 Da) was freshly prepared. Turbidity measurements were performed by adding 100 µL of the glycopolymer solution in HBS buffer to 500 µL of stock Con A solution. Final concentrations were 50 µM per mannose residue and 20 µM per Con A binding site, corresponding to a ratio Con A binding site/mannose = 1:2.5. The Con A-glycopolymer solution was vigorously stirred for 11 s using a micropipette and then placed in a Jasco V-630 UV–vis spectrometer at 30 °C, using semi-micro disposable cuvettes (optical PS, 2.5 mL volume, 1 cm path length). Optical Density (OD) data were recorded at 420 nm every 12 s for 10 min. *t*_1/2_ was calculated as the time to reach half of the maximum optical density in the OD curve. The first three points of the curve was fit with a straight line and its slope was used to determine the initial rate of Con A aggregation *k*.

## Supporting Information

File 1 Additional experimental data.
