# Supplementary material for: Mannosylated brush copolymers based on poly(ethylene glycol) and poly(ε-caprolactone) as multivalent lectin-binding nanomaterials
Source: Beilstein J Nanotechnol. 2019 Nov 7;10:2192–206. doi: 10.3762/bjnano.10.212 (PMC6880840; doi:10.3762/bjnano.10.212)
Supplement: File 1 — Additional experimental data. [file Beilstein_J_Nanotechnol-10-2192-s001.pdf]

BEILSTEIN JOURNAL OF NANOTECHNOLOGY

# Supporting Information

for

# Mannosylated brush copolymers based on poly(ethylene glycol) and poly(ε-caprolactone) as multivalent lectin-binding nanomaterials

Stefania Ordanini, Wanda Celentano, Anna Bernardi and Francesco Cellesi

*Beilstein J. Nanotechnol.* **2019,** *10,* 2192–2206. doi:10.3762/bjnano.10.212

# Additional experimental data

License and Terms: This is a supporting information file under the terms of the Creative Commons Attribution License (http://creativecommons.org/licenses/by/4.0). Please note that the reuse, redistribution and reproduction in particular requires that the authors and source are credited.
The license is subject to the *Beilstein Journal of Nanotechnology* terms and conditions: (https://www.beilstein-journals.org/bjnano)

**Table of Contents** p. S2

**Syntheses**

Synthesis of $PEG_7$-$PCL_3$ p. S3

Synthesis of TMS-propargyl-poly(ε-caprolactone) (TMS-Pg-PCL) p. S3

Synthesis of TMS-propargyl-poly(ε-caprolactone)-methacrylate (TMS-Pg-PCL-MA) p. S4

Synthesis of TMS-Pg-PCL homopolymer p. S5

Synthesis of 2-azidoethyl α-ᴅ-mannopyranoside and Figure S1 p. S5

**$^1$H NMR spectra** of:

- Figure S2: PCL-MA and selected PCL-PEG copolymers p. S7
- Figure S3: $PEG_7$-$PCL_3$ reaction mixture p. S8
- Figure S4: TMS-Pg-PCL homopolymer reaction mixture p. S9
- Figure S5: 2-azidoethyl α-D-mannopyranoside p. S9
- Figure S6: $PEG_8$-$PCL_2$-$Man_2$ p. S10
- Figure S7: A4-$PEG_8$-$PCL_2$-$Man_2$ p. S10
- Figure S8: $PEG_9$-$PCL_1$-$Man_1$ p. S11
- Figure S9: A4-$PEG_9$-$PCL_1$-$Man_1$ p. S11

**Kinetic profile**

Figure S10: Kinetic profile for the synthesis of TMS-$PEG_8$-$PCL_2$ catalyzed by CuBr/PMDETA p. S12

**DLS analyses**

Figure S11: $PEG_8$-$PCL_2$ in water/PBS/NaCl at 25 °C. p. S12

Figure S12: A4-$PEG_8$-$PCL_2$ in water/PBS/NaCl at 25 °C. p. S13

Figure S13: $PEG_9$-$PCL_1$ in water/PBS/NaCl at 25 °C. p. S13

Figure S14: A4-$PEG_9$-$PCL_1$ in water/PBS/NaCl at 25 °C. p. S14

Figure S15: $PEG_8$-$PCL_2$ in water/PBS/NaCl at 37 °C. p. S14

Figure S16: A4-$PEG_8$-$PCL_2$ in water/PBS/NaCl at 37 °C. p. S15

Figure S17: $PEG_9$-$PCL_1$ in water/PBS/NaCl at 37 °C. p. S15

Figure S18: A4-$PEG_9$-$PCL_1$ in water/PBS/NaCl at 37 °C. p. S16

Figure S19: $PEG_8$-$PCL_2$ in water/PBS/NaCl at 10 °C. p. S16

Figure S20: A4-$PEG_8$-$PCL_2$ in water/PBS/NaCl at 10 °C. p. S17

Figure S21: $PEG_9$-$PCL_1$ in water/PBS/NaCl at 10 °C. p. S17

Figure S22: A4-$PEG_9$-$PCL_1$ in water/PBS/NaCl at 10 °C. p. S18

Figure S23: $PEG_8$-$PCL_2$-$Man_2$, A4-$PEG_8$-$PCL_2$-$Man_2$, $PEG_9$-$PCL_1$-$Man_1$, A4-$PEG_9$-$PCL_1$-$Man_1$ in water at 25 °C. p. S18

Figure S24: $PEG_8$-$PCL_2$-$Man_2$, A4-$PEG_8$-$PCL_2$-$Man_2$, $PEG_9$-$PCL_1$-$Man_1$, A4-$PEG_9$-$PCL_1$-$Man_1$ in HBS at 30 °C. p. S19
Table S1: $PEG_8$-$PCL_2$ at 10, 25 or 37 °C, 1 mg/mL in $H_2O$/PBS/NaCl solution p. S19
Table S2: A4-$PEG_8$-$PCL_2$ at 10, 25 or 37 °C, 1 mg/mL in $H_2O$/PBS/NaCl solution p. S20
Table S3: $PEG_9$-$PCL_1$ at 10, 25 or 37 °C, 1 mg/mL in $H_2O$/PBS/NaCl solution. p. S21
Table S4: A4-$PEG_9$-$PCL_1$ at 10, 25 or 37 °C, 1 mg/mL in $H_2O$/PBS/NaCl solution p. S22

# Syntheses

## Synthesis of $PEG_7$-$PCL_3$

THF (inhibitor-free) was degassed under nitrogen for 10 min. Pg-PCL-MA (510 mg, 0.636 mmol, 3 equiv) and PEG-MA poly(ethylene glycol) methyl ether methacrylate (742 g, 1.484 mmol, 7 equiv) were added in a Schlenk tube and three cycles of vacuum–nitrogen were performed.

The catalyst solution was prepared as follows: Copper(I)bromide (400 mg) was added to a Schlenk tube and three cycles of vacuum–nitrogen were performed. THF (4 mL) and the ligand 1,1,4,7,10,10-hexamethyltriethylenetetramine (HMTETA, 0.76 mL) were added, obtaining a light green mixture that was stirred at room temperature under $N_2$ for 10 min.

Finally, THF (4.12 mL), the catalyst solution (0.30 mL, containing CuBr 30 mg, 0.212 mmol, 1 equiv and HMTETA 0.058 mL, 0.212 mmol, 1 equiv) and ethyl 2-bromo-2-methylpropionate (31 µL, 0.212 mmol, 1 equiv) as initiator were added to the monomer solution; the reaction mixture was stirred for 6 h at 50 °C under nitrogen atmosphere; conversion=58%.

## Synthesis of TMS-propargyl-poly(ε-caprolactone) (TMS-Pg-PCL)

ε-caprolactone (9 g, 0.079 mol, 5 equiv) was added to a round flask and heated up to 130 °C. A second yellowish solution containing 3-(trimethylsilyl)propargyl alcohol (2.34 mL, 0.016 mol, 1 equiv) and tin(II) 2-ethylhexanoate ($Sn(Oct)_2$) (32 mg, 0.0788 mmol, 0.005 equiv) was prepared, stirred under a nitrogen flow for 20 min, and then added to the ε-caprolactone.

The reaction mixture was stirred for 3 h at 130 °C, then the reaction was stopped by cooling the flask to room temperature. Purification was carried out by dissolving the crude in 3 mL of methanol and dripping it in 180 mL of vigorously stirred water. The resulting precipitate was isolated by removing the supernatant, dissolved in $CH_2Cl_2$ and anhydrified with anhydrous sodium sulfate. Subsequently it was filtered, and the filtrate was dried under reduced pressure, obtaining 2.9 g of final product TMS-Pg-PCL as a colorless viscous oil. $\text{Conversion}_{\varepsilon\text{-CL}}$ = 97%; $\text{conversion}_{\text{propargyl alcohol}}$ = 95%; yield = 97%; $M_{n,NMR}$ = 770.9 g·mol$^{-1}$. $^1$H NMR (400 MHz, $CDCl_3$) δ 4.62 (s, 2H, -C$H_2$-CC-TMS), 4.01 (t, $J$ = 6.7 Hz, 2H·($n$ − 1), -C$H_2$-OC(O)-), 3.59 (t, $J$ = 6.5 Hz, 2H, -C$H_2$-OH), 2.38–2.17 (m, 2H·$n$, -OC(O)-C$H_2$-), 1.72–1.45 (m, 4H·$n$, -OC(O)-$CH_2$C$H_2$-, -C$H_2$$CH_2$-OC(O)-), 1.41–1.25 (m, 2H·$n$, -$CH_2$C$H_2$$CH_2$-), 0.14 (s, 9H, -$CH_2$-CC-*TMS*).

## Synthesis of TMS-propargyl-poly(ε-caprolactone)-methacrylate (TMS-Pg-PCL-MA)

TMS-Pg-PCL (6.3 g, 0.0082 mol, 1 equiv) was added in a round flask and three cycles of vacuum–nitrogen were performed. 210 mL of dry toluene were added under nitrogen flow and the flask was cooled to 0 °C for 30 min. Triethylamine (1.71 mL, 0.0122 mol, 1.5 equiv) and freshly distilled methacryloyl chloride (1.02 mL, 0.0122 mol, 1.5 equiv) were sequentially added to the reaction mixture. The reaction was stirred at room temperature under dynamic nitrogen atmosphere for 15 min and under static nitrogen atmosphere overnight. The crude was filtered through a celite pad ($h$ = 6 cm, Φ = 2 cm), washing with toluene, and then the filtrate was evaporated under reduced pressure. The residue was taken up in 140 mL of $CH_2Cl_2$, washed with brine (3 × 55 mL) and dried over anhydrous sodium sulfate. The solvent was evaporated under vacuum, obtaining 5.25 g of final product TMS-Pg-PCL-MA as a pale yellow viscous oil. $\text{Conversion}_{\text{OH->OMA}}$ = 95%; yield = 76%; $M_{n,NMR}$ = 818.7 g·mol$^{-1}$. $^1$H NMR (400 MHz, $CDCl_3$) δ 6.04 (s, 1H, $H_2$C-C($CH_3$)-), 5.50 (s, 1H, $H_2$C-C($CH_3$)-), 4.63 (s, 2H, -C$H_2$-

CC-TMS), 4.10 (t, $J$ = 6.6 Hz, 2H, -C$H_2$-O-MA), 4.02 (t, $J$ = 6.7 Hz, 2H·($n$ − 1), -C$H_2$-OC(O)-), 2.40–2.18 (m, 2H·$n$, -OC(O)-C$H_2$-), 1.89 (s, 3H, $H_2$C-C(C$H_3$)-), 1.70–1.50 (m, 4H·$n$, -OC(O)-$CH_2$C$H_2$-, -C$H_2$$CH_2$-OC(O)-), 1.42–1.24 (m, 2H·$n$, -$CH_2$C$H_2$$CH_2$-), 0.14 (s, 9H, -$CH_2$-CC-*TMS*).

## Synthesis of TMS-Pg-PCL homopolymer

THF (inhibitor-free) was degassed under nitrogen for 10 min. TMS-Pg-PCL-MA (1093 mg, 1.3 mmol, 10 equiv) was added in a Schlenk tube and three cycles of vacuum–nitrogen were performed. The catalyst solution was prepared as follows: Copper(I)bromide (500 mg) was added to a Schlenk tube and three cycles of vacuum–nitrogen were performed. THF (6 mL) and the ligand 1,1,4,7,10,10-hexamethyltriethylenetetramine (HMTETA, 0.95 mL) were added, obtaining a light green mixture that was stirred at room temperature under $N_2$ for 10 min. Finally, THF (1.776 mL), the catalyst solution (0.224 mL, containing CuBr 18.65 mg, 0.13 mmol, 1 equiv and HMTETA 0.035 mL, 0.13 mmol, 1 equiv) and ethyl 2-bromo-2-methylpropionate (19 µL, 0.13 mmol, 1 equiv) as initiator were added to the monomer solution; the reaction mixture was stirred for 7 h at 50 °C under nitrogen atmosphere; conversion = 55%.

## Synthesis of the azide-functionalized mannose

The synthesis of (2-azidoethyl)-α-ᴅ-mannopyranoside (**3**) was performed starting from penta-*O*-acetyl mannose (**1**), according to [1] (Figure S1). Briefly, **1** first reacted with chloroethanol in the presence of boron trifluoride diethyl etherate as activator, to give (2-chloroethyl)-2,3,4,6-tetraacetyl-α-ᴅ-mannopyranoside (**2-Cl**). A chloride–azide exchange reaction catalyzed by tetrabutylammonium iodide led to (2-azidoethyl)-2,3,4,6-tetraacetyl-α-ᴅ-mannopyranoside (**2-N$_3$**). Finally, the acetate protecting groups were removed by Zemplen deprotection; 125 mg of the final product were obtained with 25% overall yield. The spectral data of the resulting compound were in agreement with those reported in the literature. $^1$H NMR (400 MHz, $D_2O$)

δ 4.93 (s, 1H, $H_1$), 3.99 (s, 1H, $H_2$), 3.98–3.87 (m, 2H, $H_{7B}$, $H_{6B}$), 3.85 (dd, *J* = 8.5, 2.8 Hz, 1H, $H_3$), 3.82–3.74 (m, 2H, $H_{7A}$, $H_{6A}$), 3.73–3.63 (m, 2H, $H_4$, $H_5$), 3.60–3.45 (m, 2H, $H_8$).

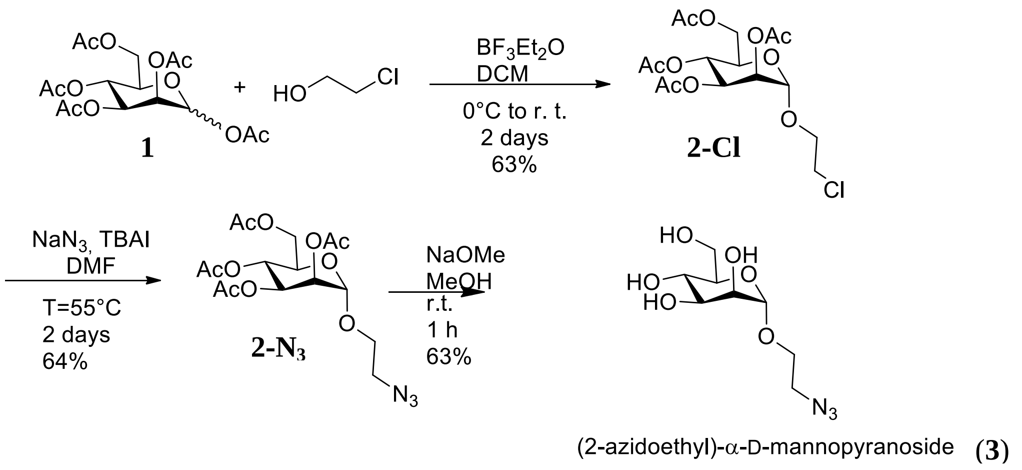


**Figure S1:** Synthetic pathway for the synthesis of (2-azidoethyl)-α-D-mannopyranoside (**3**) starting from penta-*O*-acetyl mannose (**1**).

# $^1$H NMR spectra

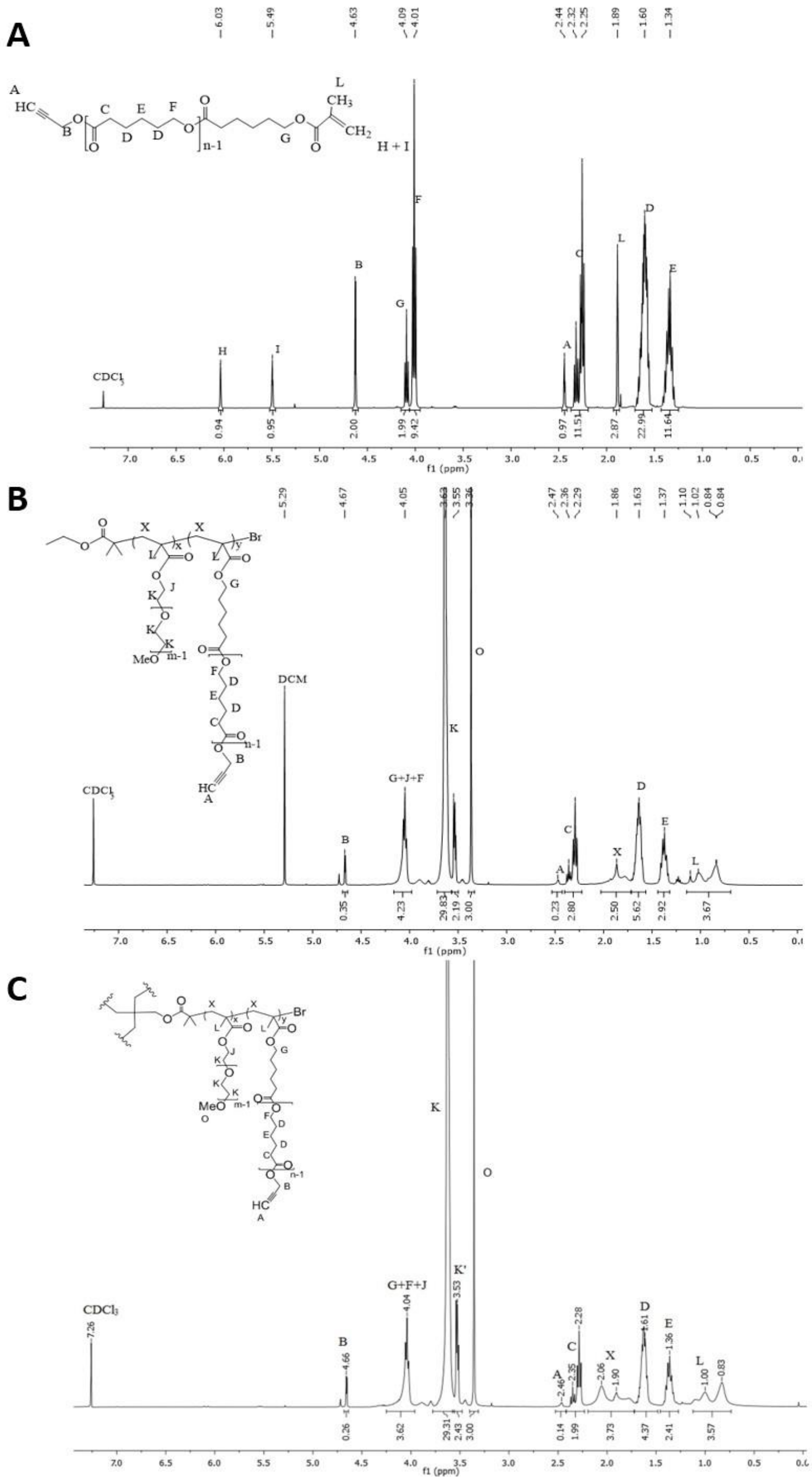

**Figure S2:** $^1$H NMR ($CDCl_3$) spectra of A) PCL-MA starting material, B) $PEG_8$-$PCL_2$ and C) A4-$PEG_8$-$PCL_2$ copolymer. The final copolymer composition was verified by calculating the ratio between the number of PEG repeating units and the total number of repeating units in the polymer chains; it is represented by the ratio of the peaks “O” and “L”, which correspond to the three protons of the terminal $CH_3$- group of the PEG macromonomer, and the $CH_3$- group of the methacrylate backbone of the copolymer, respectively.

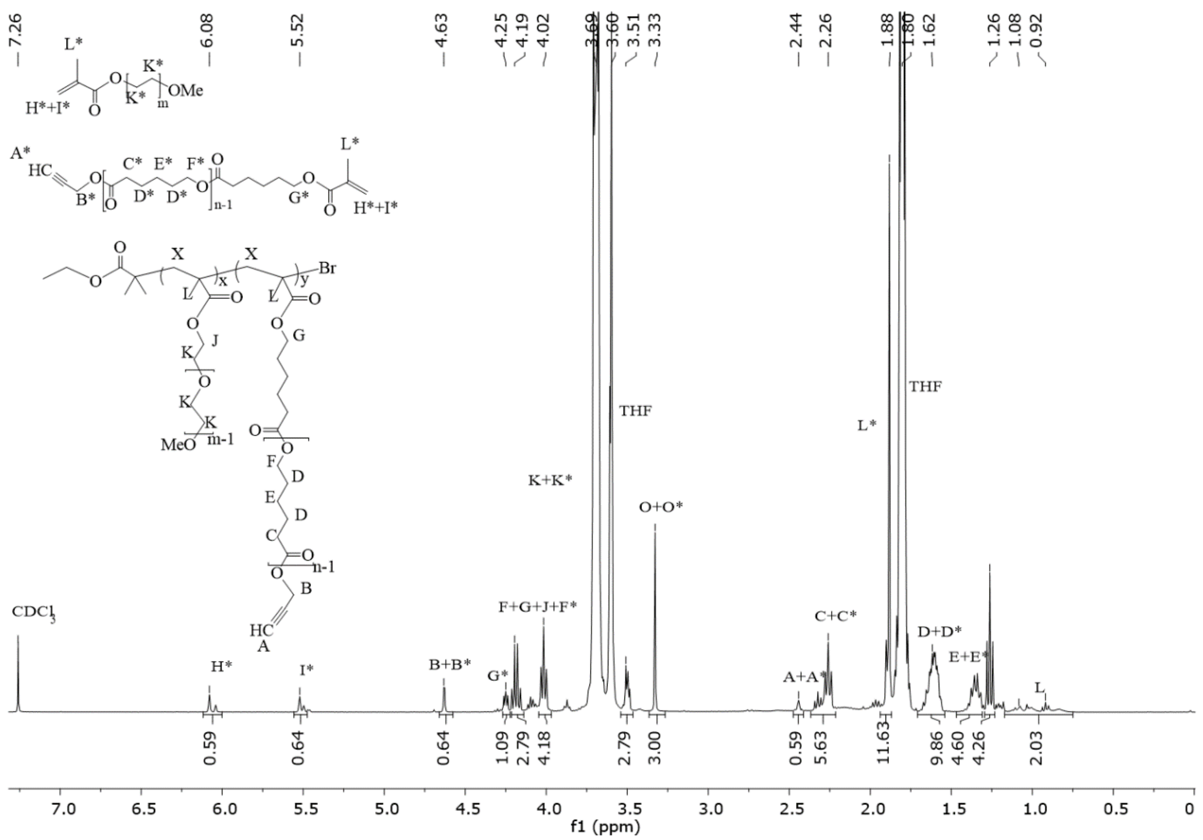


**Figure S3:** $^1$H NMR ($CDCl_3$) spectrum of the reaction mixture to obtain $PEG_7$-$PCL_3$ (after 6 h). Conversion values were obtained by peaks ‘‘H’’ and ‘‘I’’, corresponding to the two protons on the double bond of the reagents: They were set as reference and compared with peak L, which represents the methyl groups of the product.

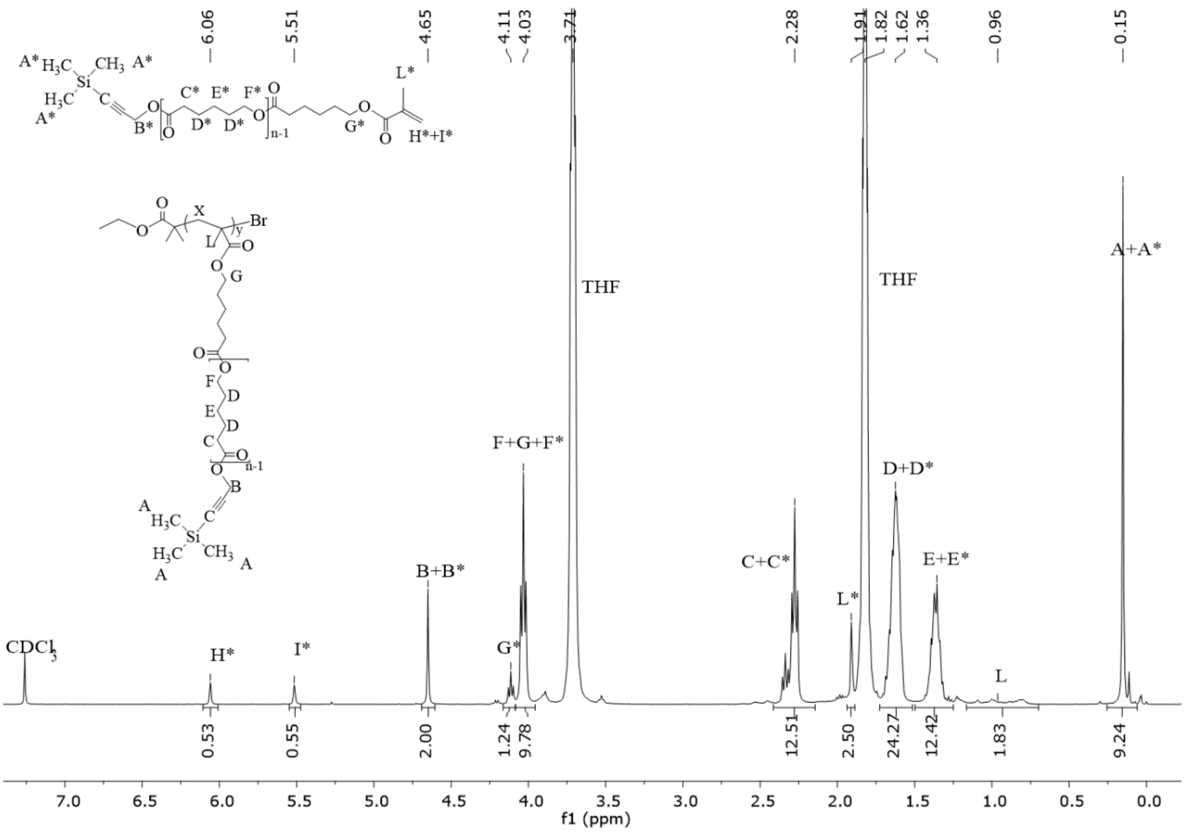


**Figure S4:** $^{1}$H NMR ($CDCl_3$) spectrum of the reaction mixture to obtain TMS-Pg-PCL-MA homopolymer (6 h).

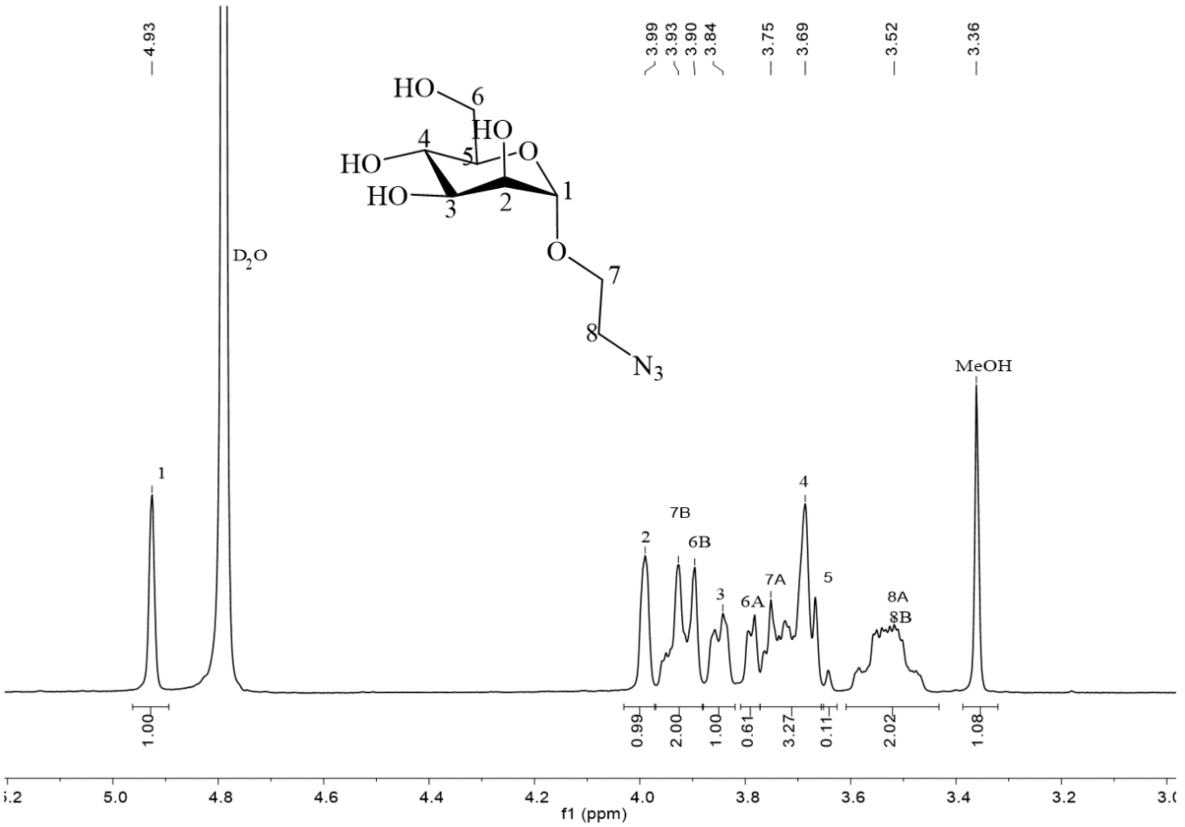


**Figure S5:** $^{1}$H NMR ($D_2O$) spectrum of (2-azidoethyl)-α-D-mannopyranoside.

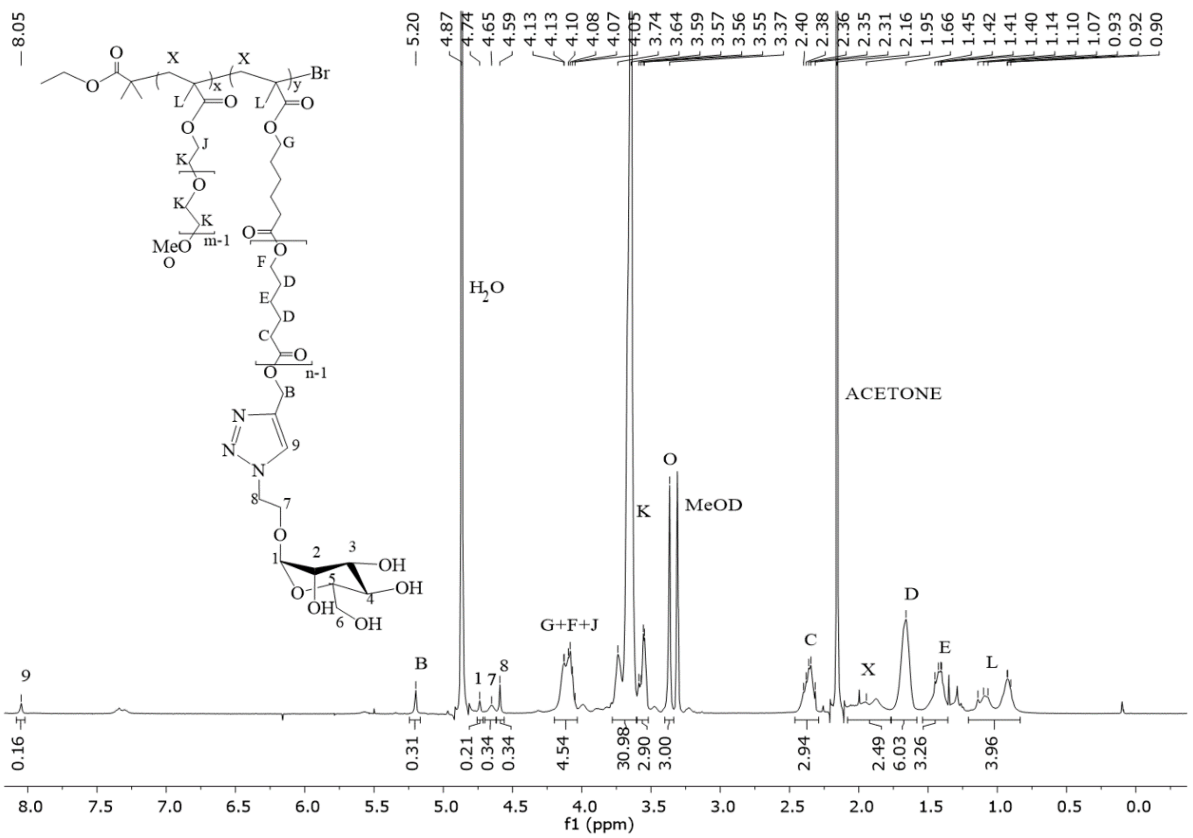


**Figure S6:** $^{1}$H NMR (MeOD) spectrum of divalent glycopolymer PEG$_8$-PCL$_2$-Man$_2$.

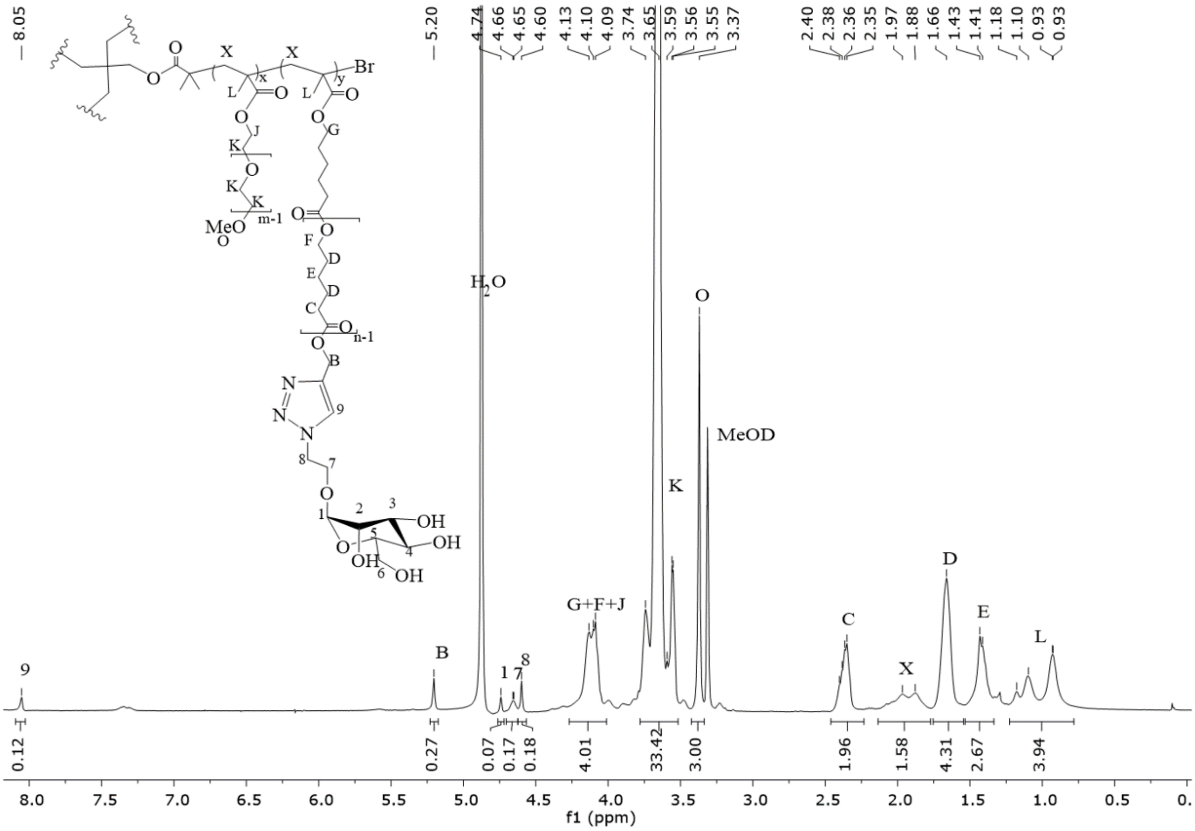


**Figure S7:** $^{1}$H NMR (MeOD) spectrum of octavalent glycopolymer A4-PEG$_8$-PCL$_2$-Man$_2$.

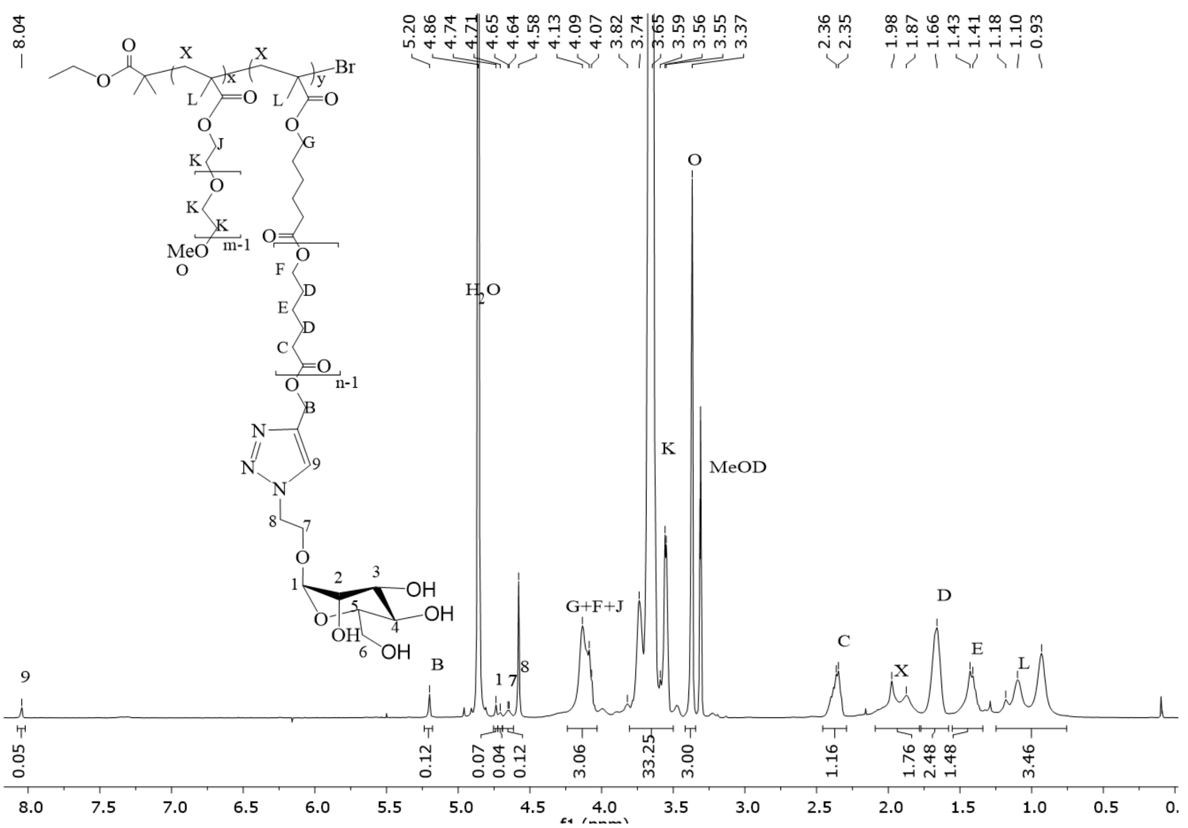

**Figure S8:** $^1$H NMR (MeOD) spectrum of monovalent glycopolymer PEG$_9$-PCL$_1$-Man$_1$.

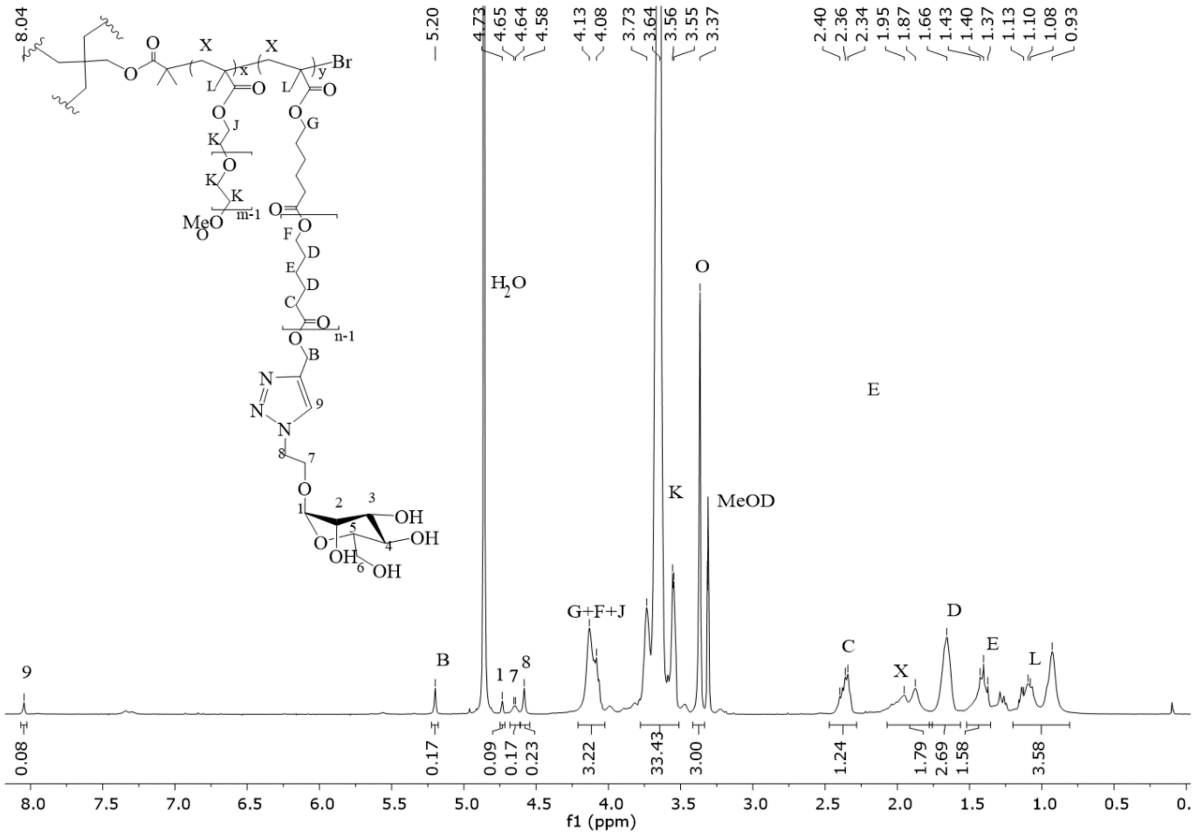

**Figure S9:** $^1$H NMR (MeOD) spectrum of tetravalent glycopolymer A4-PEG$_9$-PCL$_1$-Man$_1$.

# Kinetic profile

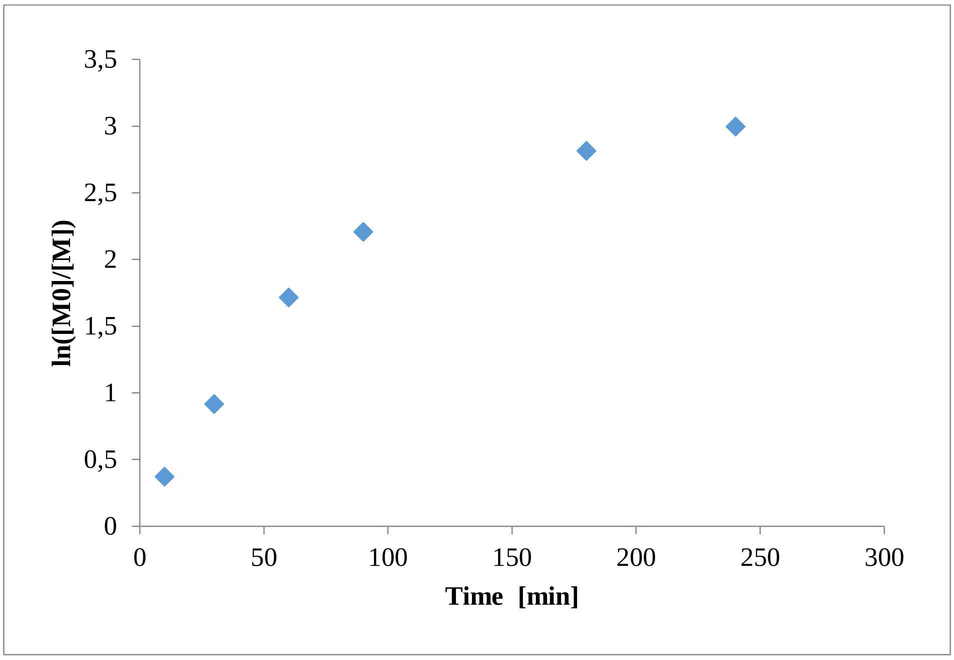


**Figure S10:** Kinetic profile obtained for the synthesis of TMS-PEG$_8$-PCL$_2$. The reaction was conducted at 50 °C under nitrogen atmosphere for 6 h, using CuBr/PMDETA as catalytic system.

# DLS analyses

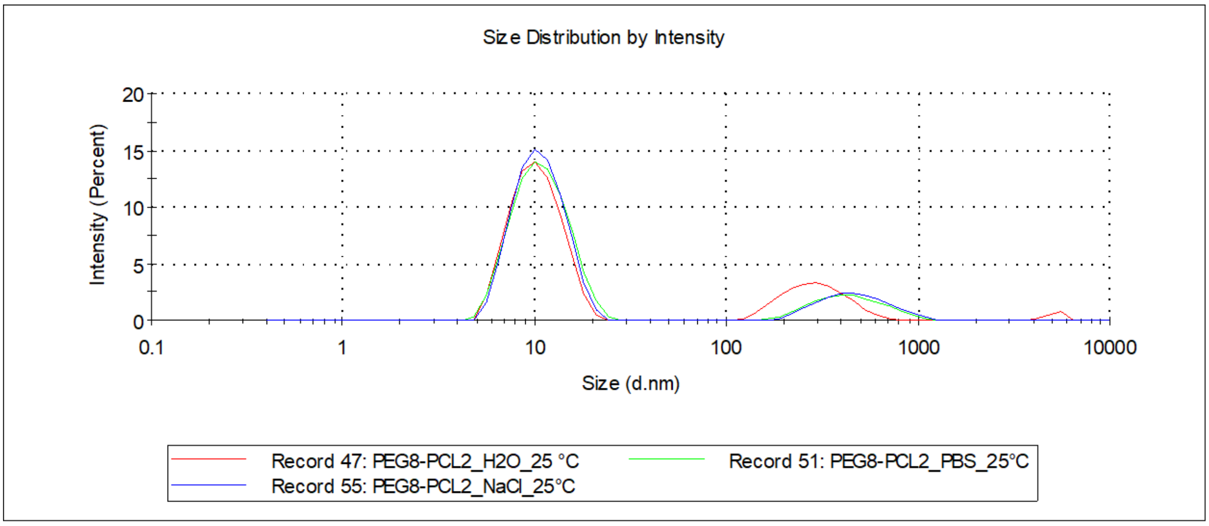


**Figure S11:** Size distribution by intensity of PEG$_8$-PCL$_2$; 1 mg/mL in water, PBS or NaCl (0.9% w/v) at 25 °C, filtered samples (PTFE 0.45 µm).

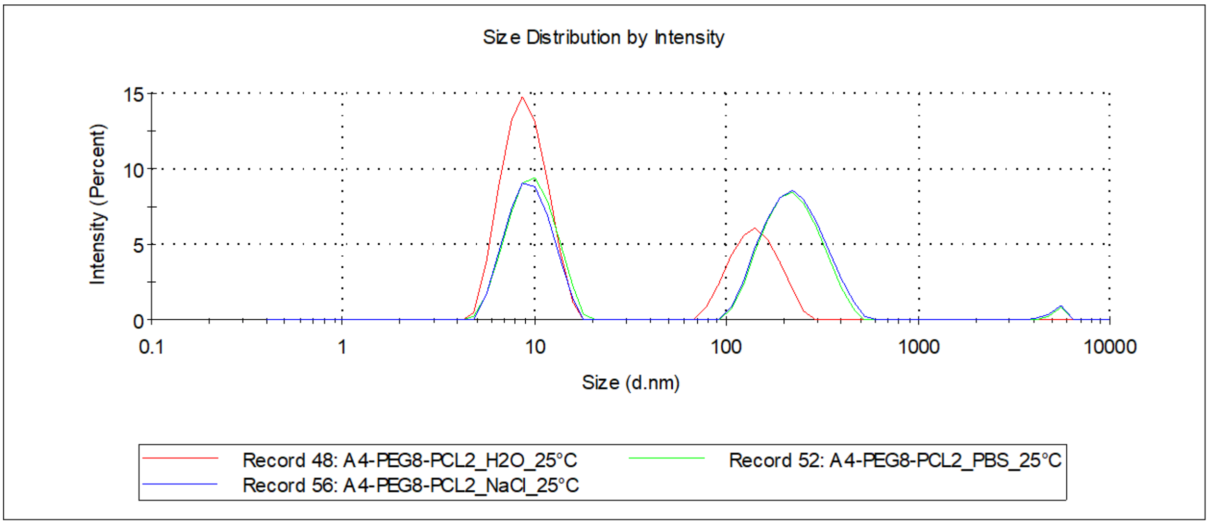


**Figure S12:** Size distribution by intensity of A4-$PEG_8$-$PCL_2$; 1 mg/mL in water, PBS or NaCl (0.9% w/v) at 25 °C, filtered samples (PTFE 0.45 µm).

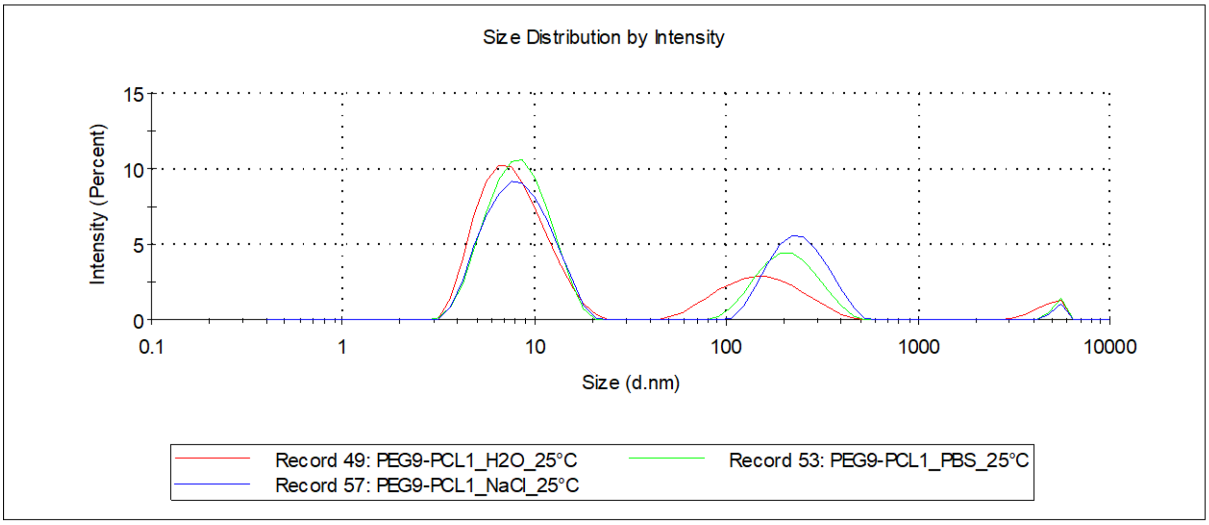


**Figure S13:** Size distribution by intensity of $PEG_9$-$PCL_1$; 1 mg/mL in water, PBS or NaCl (0.9% w/v) at 25 °C, filtered samples (PTFE 0.45 µm).

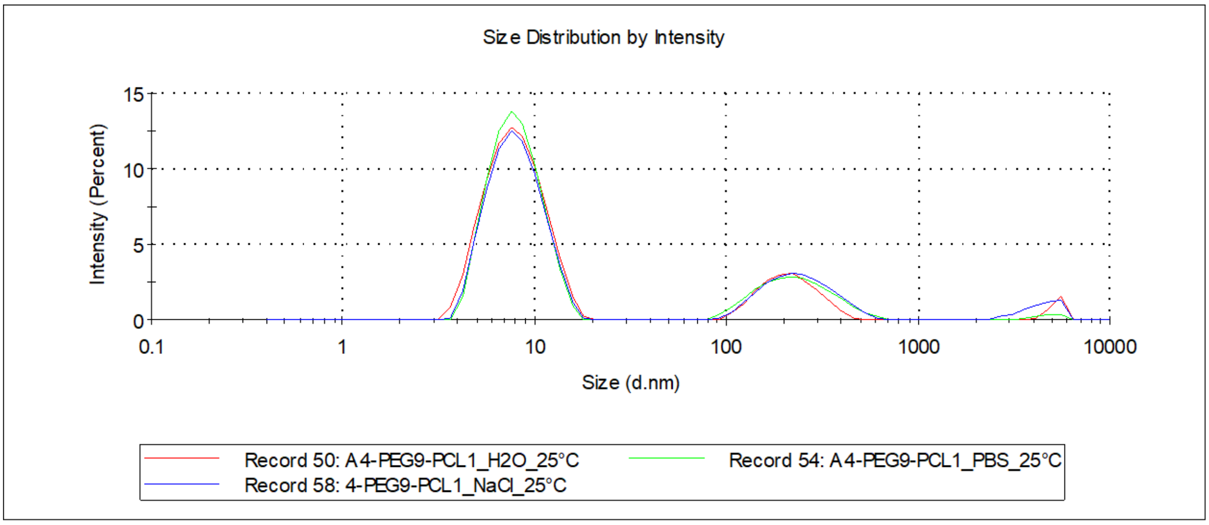


**Figure S14:** Size distribution by intensity of A4-$PEG_9$-$PCL_1$; 1 mg/mL in water, PBS or NaCl (0.9% w/v) at 25 °C, filtered samples (PTFE 0.45 µm).

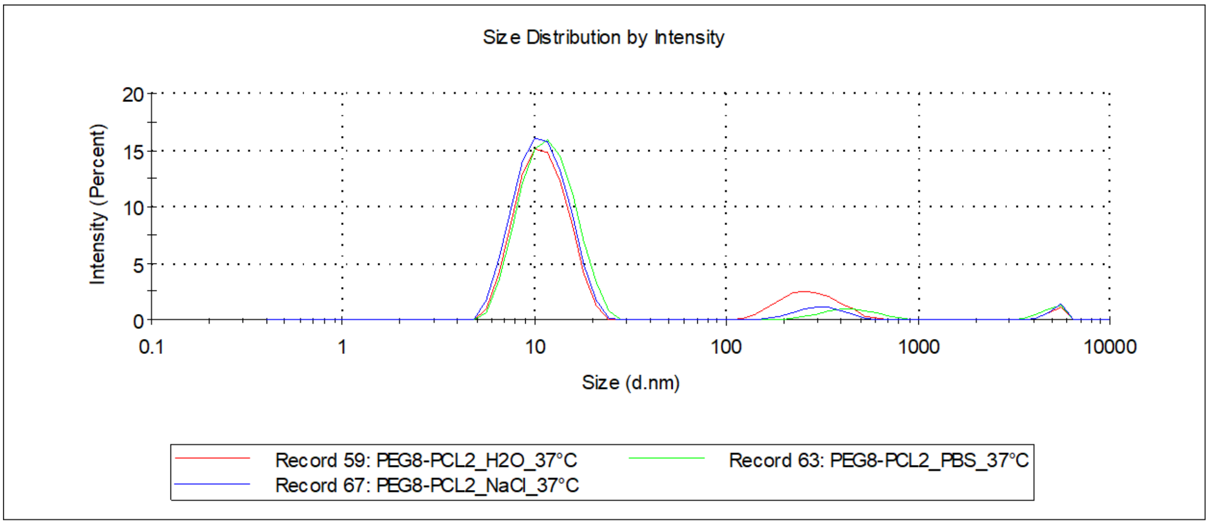


**Figure S15:** Size distribution by intensity of $PEG_8$-$PCL_2$; 1 mg/mL in water, PBS or NaCl (0.9% w/v) at 37 °C, filtered samples (PTFE 0.45 µm).

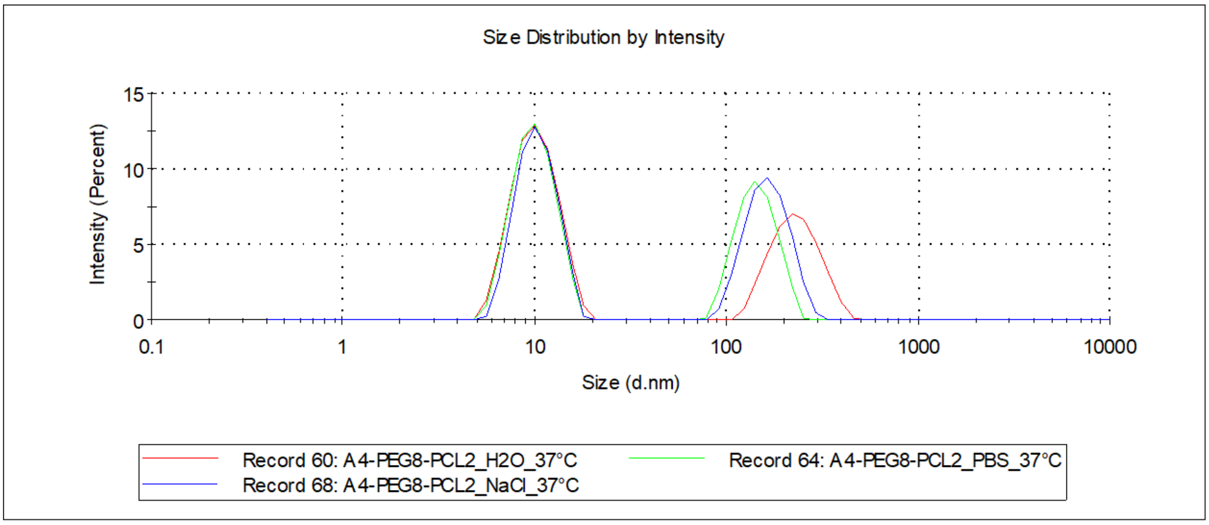


**Figure S16:** Size distribution by intensity of A4-$PEG_8$-$PCL_2$; 1 mg/mL in water, PBS or NaCl (0.9% w/v) at 37 °C, filtered samples (PTFE 0.45 µm).

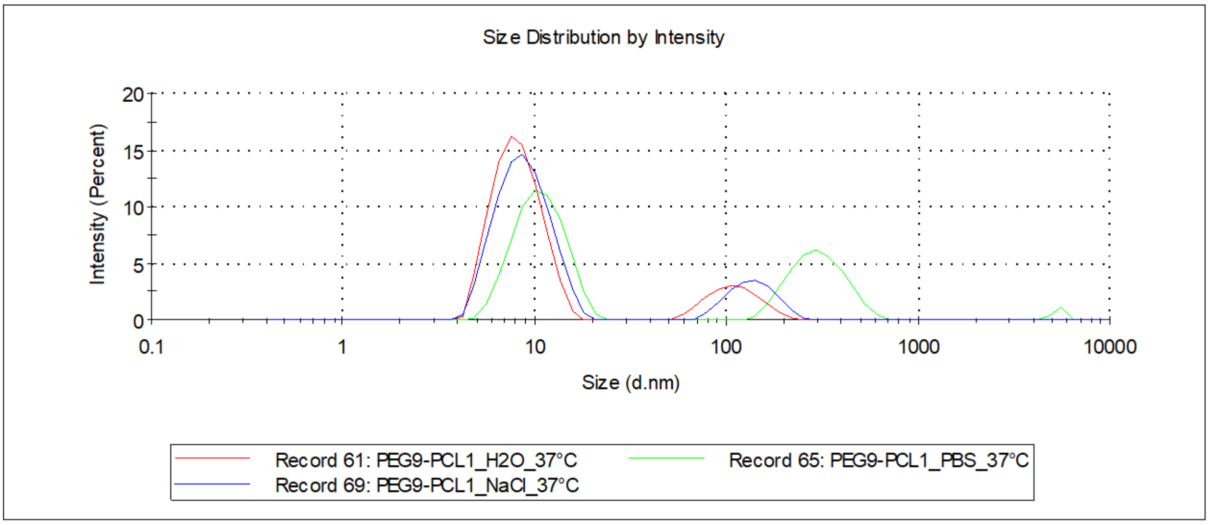


**Figure S17:** Size distribution by intensity of $PEG_9$-$PCL_1$; 1 mg/mL in water, PBS or NaCl (0.9% w/v) at 37 °C, filtered samples (PTFE 0.45 µm).

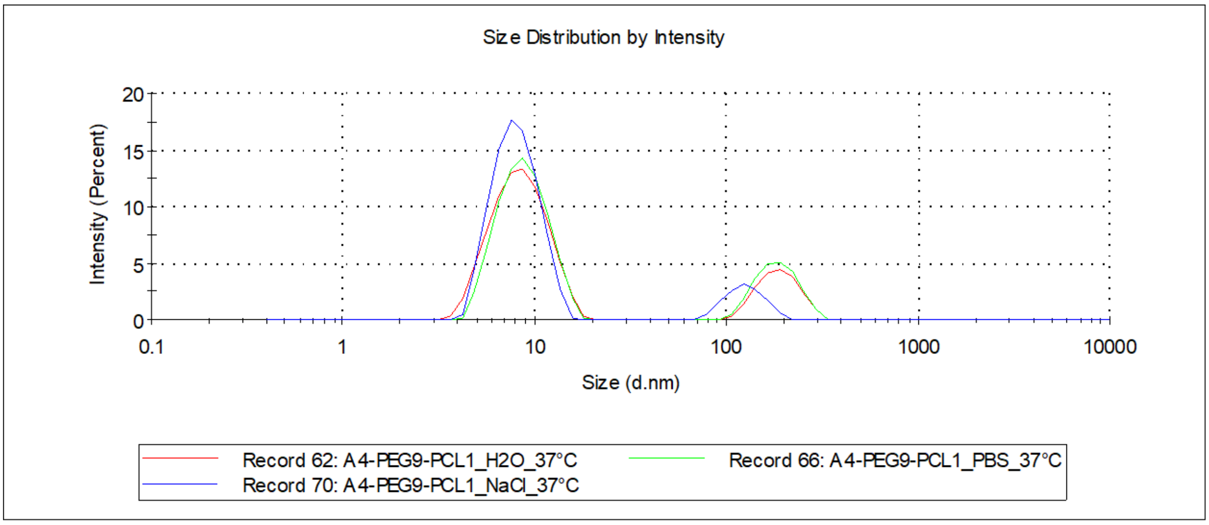


**Figure S18:** Size distribution by intensity of A4-PEG$_9$-PCL$_1$; 1 mg/mL in water, PBS or NaCl (0.9% w/v) at 37 °C, filtered samples (PTFE 0.45 µm).

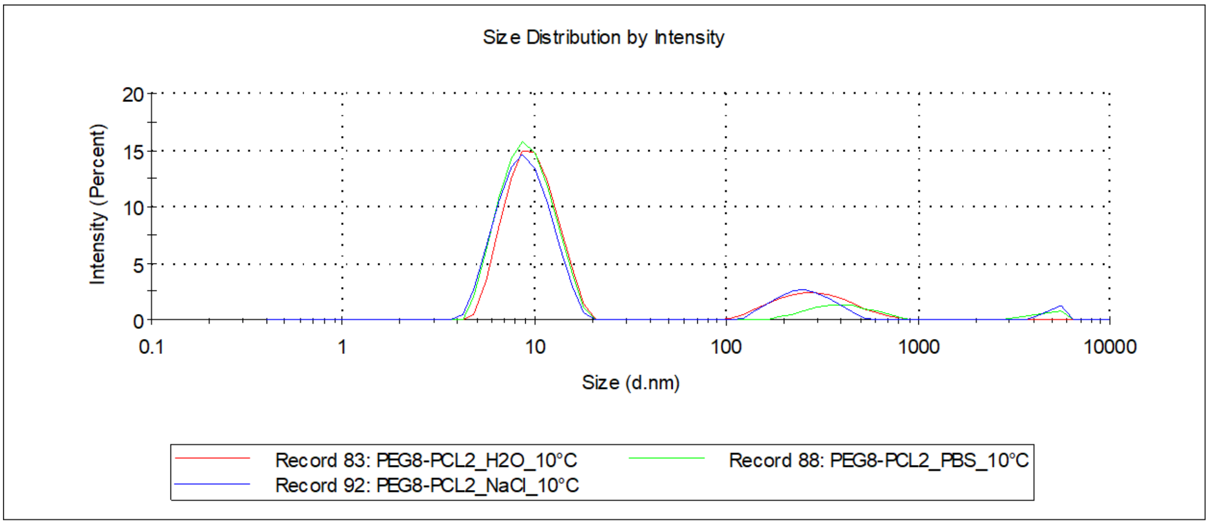


**Figure S19:** Size distribution by intensity of PEG$_8$-PCL$_2$; 1 mg/mL in water, PBS or NaCl (0.9% w/v) at 10 °C, filtered samples (PTFE 0.45 µm).

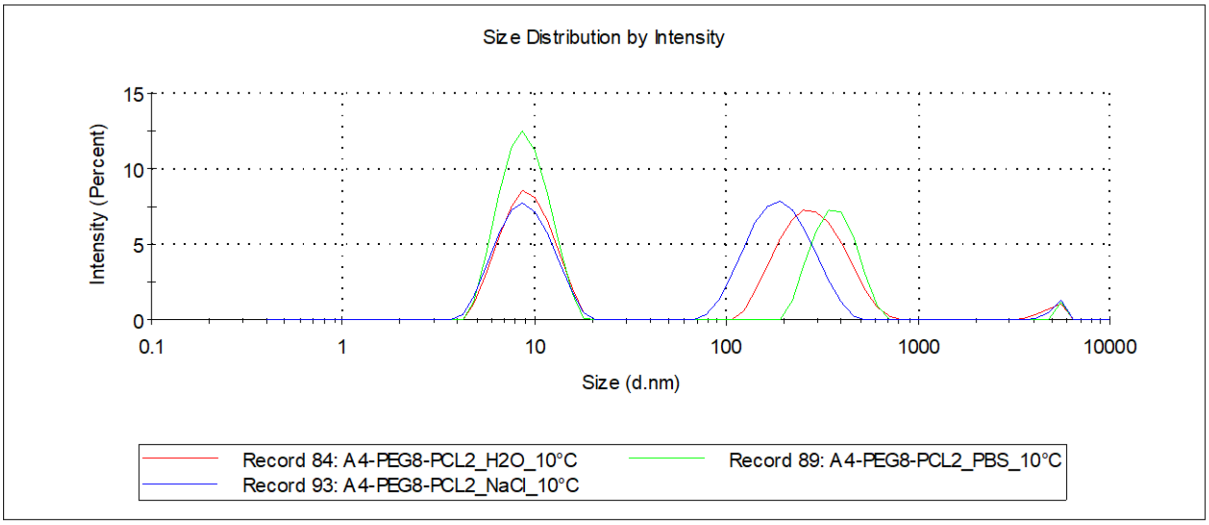


**Figure S20:** Size distribution by intensity of A4-PEG$_8$-PCL$_2$; 1 mg/mL in water, PBS or NaCl (0.9% w/v) at 10 °C, filtered samples (PTFE 0.45 µm).

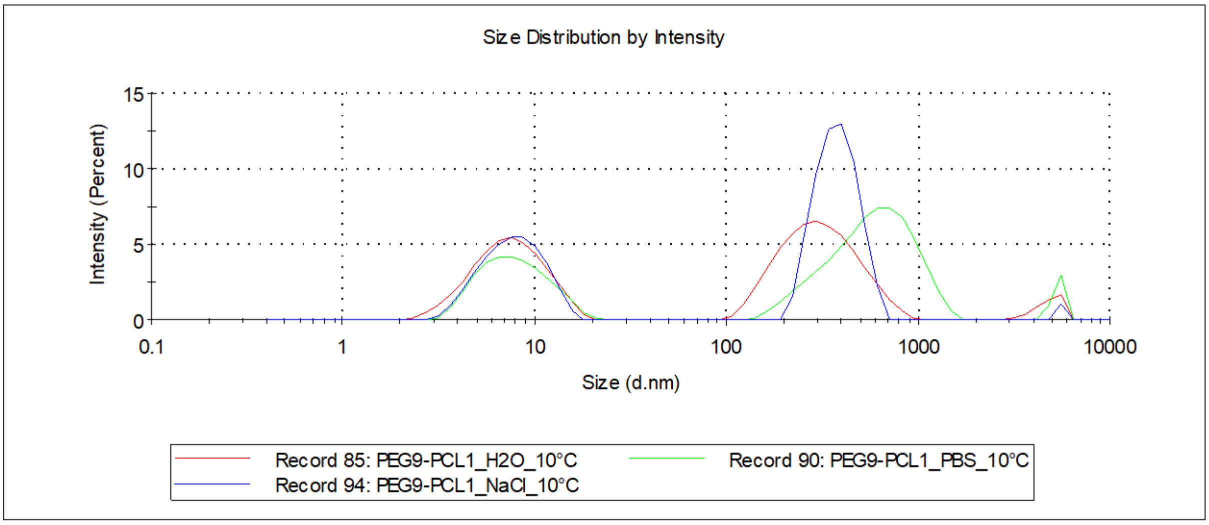


**Figure S21:** Size distribution by intensity of PEG$_9$-PCL$_1$; 1 mg/mL in water, PBS or NaCl (0.9% w/v) at 10 °C, filtered samples (PTFE 0.45 µm).

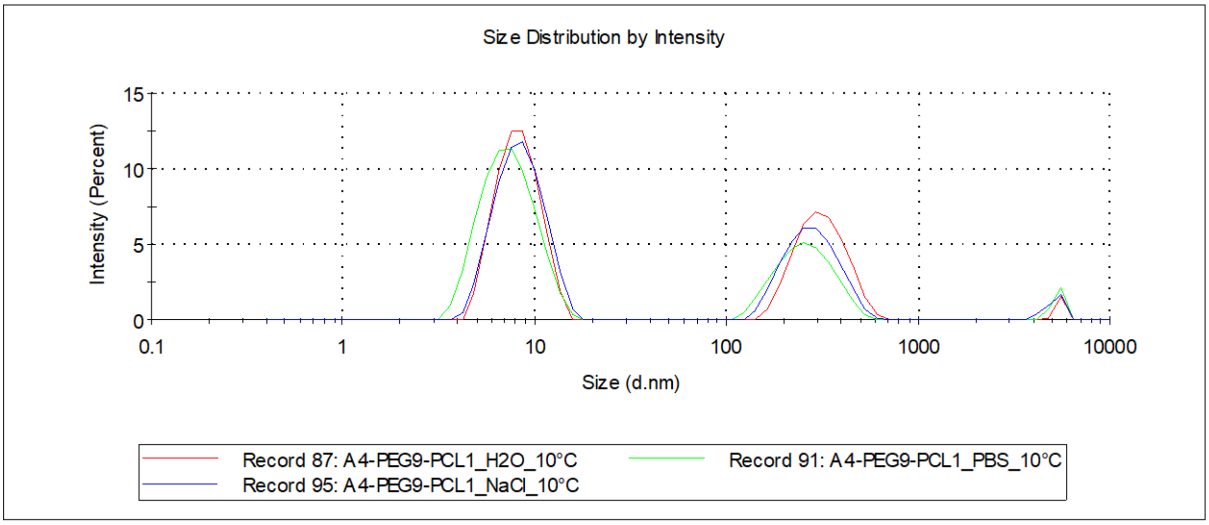


**Figure S22:** Size distribution by intensity of A4-PEG$_9$-PCL$_1$; 1 mg/mL in water, PBS or NaCl (0.9% w/v) at 10 °C, filtered samples (PTFE 0.45 µm).

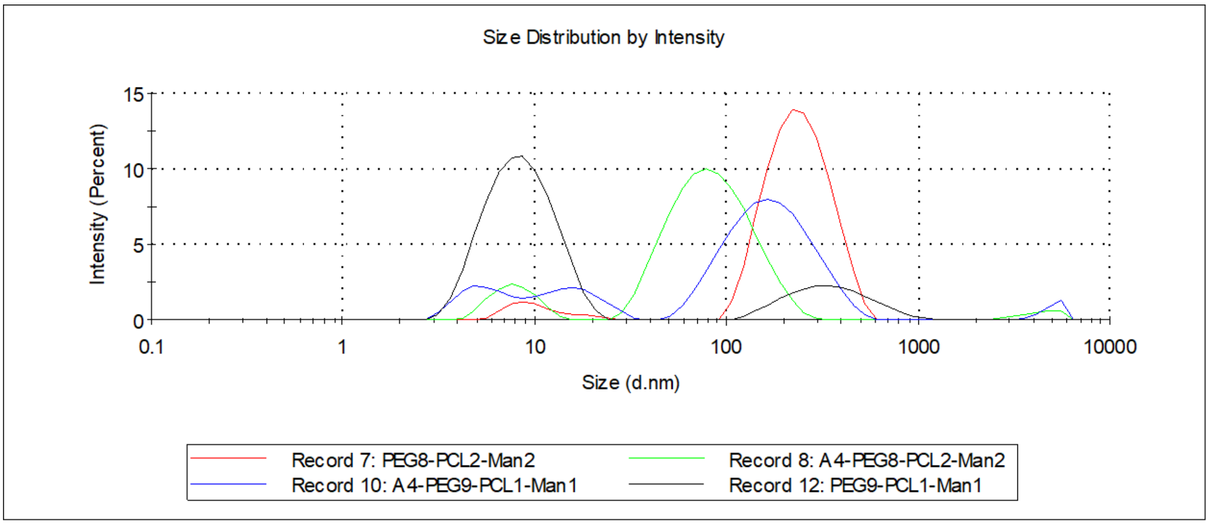


**Figure S23:** Size distribution by intensity of PEG$_8$-PCL$_2$-Man$_2$, A4-PEG$_8$-PCL$_2$-Man$_2$, PEG$_9$-PCL$_1$-Man$_1$, A4-PEG$_9$-PCL$_1$-Man$_1$; 1 mg/mL in water at 25 °C, filtered samples (PTFE 0.45 µm).

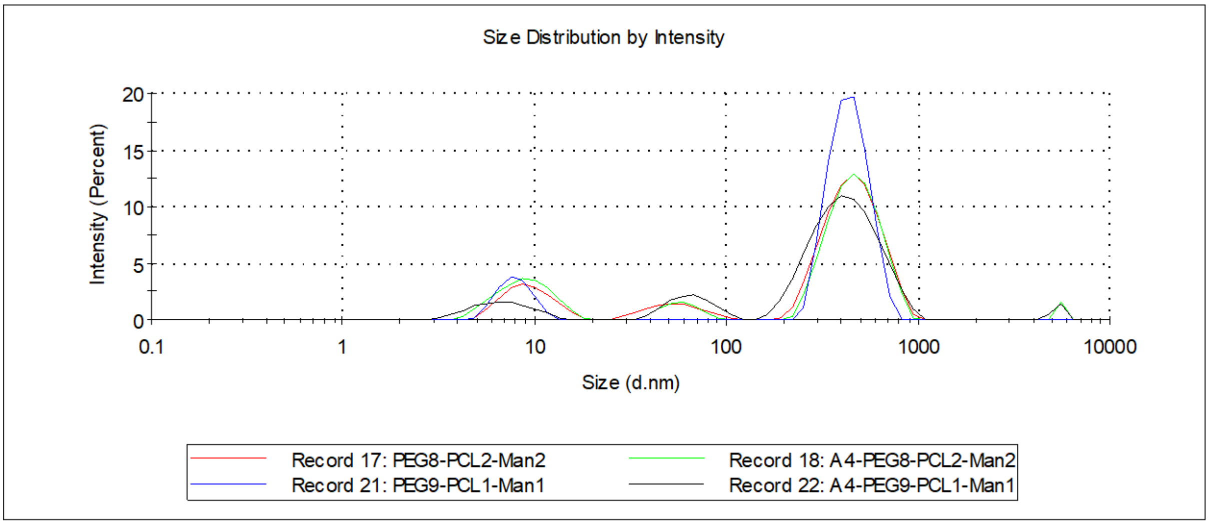


**Figure S24:** Size distribution by intensity of PEG$_8$-PCL$_2$-Man$_2$, A4-PEG$_8$-PCL$_2$-Man$_2$, PEG$_9$-PCL$_1$-Man$_1$, A4-PEG$_9$-PCL$_1$-Man$_1$; 1 mg/mL in HBS at 30 °C, filtered samples (PTFE 0.45 µm).

**Table S1:** DLS analyses of PEG$_8$-PCL$_2$ at 10, 25 or 37 °C, 1 mg/mL in $H_2O$, PBS or NaCl (0.9% w/v).

| | **PEG$_8$-PCL$_2$** | | |
|---|---|---|---|
| | **10 °C** | **25 °C** | **37 °C** |
| | $D_h$ [nm] (intensity%) | $D_h$ [nm] (intensity%) | $D_h$ [nm] (intensity%) |
| **$H_2O$** | 10.72 (72.3%)<br>361.57 (27.7% ) | 10.82 (73%)<br>271.4 (28.4%) | 11.42 (79.5%)<br>363.37 (19.4%) |
| **PBS** | 9.99 (87.2%)<br>491.43 (11.2%) | 11.26 (83.9%)<br>505.0 (16.1%) | 12.07 (91.4%)<br>478.63 (5.7%) |
| **NaCl** | 10.53 (74.9%)<br>387.43 (23.9%) | 10.88 (84.5%)<br>520.0 (15.2%) | 11.33 (94.3%)<br>262.83 (4.1%) |

**Table S2:** DLS analyses of A4-$PEG_8$-$PCL_2$ at 10, 25 or 37 °C, 1 mg/mL in $H_2O$, PBS or NaCl (0.9% w/v).

| | **A4-$PEG_8$-$PCL_2$** | | |
|---|---|---|---|
| | **10 °C** | **25 °C** | **37 °C** |
| | $D_h$ [nm] (intensity%) | $D_h$ [nm] (intensity%) | $D_h$ [nm] (intensity%) |
| **$H_2O$** | 8.44 (53.5%)<br>217.1 (44.2%) | 9.57 (62.7%)<br>196.8 (33.7%) | 10.26 (66.5%)<br>197.5 (33.4%) |
| **PBS** | 8.38 (66.9%)<br>299.13 (31.65%) | 9.77 (52.3%)<br>222.1 (46.6%) | 10.25 (56.6%)<br>172.87 (43.4%) |
| **NaCl** | 8.90 (44.7%)<br>199.43 (53.23%) | 9.52 (46%)<br>210.67 (52.5%) | 10.20 (56%)<br>168.0 (44%) |

**Table S3:** DLS analyses of $PEG_9$-$PCL_1$ at 10, 25 or 37 °C, 1 mg/mL in $H_2O$, PBS or NaCl (0.9% w/v).

| | **$PEG_9$-$PCL_1$** | | |
|---|---|---|---|
| | **10 °C** | **25 °C** | **37 °C** |
| | $D_h$ [nm] (intensity%) | $D_h$ [nm] (intensity%) | $D_h$ [nm] (intensity%) |
| **$H_2O$** | 7.51 (41.3%)<br>303.87 (56.5%) | 8.49 (72.7%)<br>192.6 (25.1%) | 9.21 (80%)<br>150.0 (18.3%) |
| **PBS** | 8.13 (30%)<br>653.6 (66%) | 8.75 (67.7%)<br>254.9 (29.8%) | 10.76 (61.4%)<br>286.73 (36.7%) |
| **NaCl** | 8.32 (33.6%)<br>444.2 (66%) | 8.97 (61.3%)<br>310.0 (37.4%) | 8.91 (82.6%)<br>138.55 (17.4%) |

**Table S4:** DLS analyses of A4-$PEG_9$-$PCL_1$ at 10, 25 or 37 °C, 1 mg/mL in $H_2O$, PBS or NaCl (0.9% w/v).

| | **A4-$PEG_9$-$PCL_1$** | | |
|---|---|---|---|
| | **10 °C** | **25 °C** | **37 °C** |
| | $D_h$ [nm] (intensity%) | $D_h$ [nm] (intensity%) | $D_h$ [nm] (intensity%) |
| **$H_2O$** | 8.14 (58.9%)<br>346.4 (38.1%) | 8.11 (79.3%)<br>242 (19%) | 8.34 (84.4%)<br>161.5 (15.6%) |
| **PBS** | 7.99 (61.8%)<br>290.4 (36.7%) | 8.01 (78%)<br>227.0 (22%) | 9.02 (73.9%)<br>209.13 (26.2%) |
| **NaCl** | 8.39 (62.6%)<br>278.87 (35.1%) | 7.93 (75.5%)<br>206.7 (22.2%) | 8.42 (81.4%)<br>168.6 (18.6%) |

# References


1. Gu, L.; Luo, P. G.; Wang, H.; Meziani, M. J.; Lin, Y.; Veca, L. M.; Cao, L.; Lu, F.; Wang, X.; Quinn, R. A.; Wang, W.; Zhang, P.; Lacher, S.; Sun, Y.-P. *Biomacromolecules* **2008**, *9*, 2408–2418. doi:10.1021/bm800395e
